# Characterization of Circular RNA Expression Profiles in Colon Specimens of Patients with Slow Transit Constipation

**DOI:** 10.1155/2022/3653363

**Published:** 2022-06-10

**Authors:** Changlei Xi, Yuntian Hong, Baoxiang Chen, Xiaoyu Xie, Weicheng Liu, Qun Qian, Congqing Jiang, Xianghai Ren

**Affiliations:** ^1^Department of Colorectal and Anal Surgery, Zhongnan Hospital of Wuhan University, Wuhan 430071, China; ^2^Department of Colorectal and Anal Surgery, The Second Clinical Medical College, Yangtze University, Jingzhou Central Hospital, Jingzhou 434020, China; ^3^Clinical Center of Intestinal and Colorectal Diseases of Hubei Province, Wuhan 430071, China; ^4^Key Laboratory of Intestinal and Colorectal Diseases of Hubei Province, Wuhan 430071, China; ^5^Colorectal and Anal Disease Research Centre, Medical School of Wuhan University, Wuhan 430071, China; ^6^Quality Control Centre of Colorectal and Anal Surgery of Health Commission of Hubei Province, Wuhan 430071, China

## Abstract

**Background:**

Slow transit constipation (STC) is a clinical syndrome characterized by a decreased urge to defecate and delayed colonic transit. Circular RNAs (circRNAs) are a recently discovered class of regulatory RNAs that have emerged as critical biomarkers and regulators of various diseases. However, the expression profiles and mechanisms underlying circRNA regulation in human STC tissues have not been explored.

**Methods:**

High-throughput RNA sequencing technology was used to compare the differences in circRNA expression profiles in colon samples taken from patients with STC or controls. Bioinformatics analyses were performed on the host genes of the differentially expressed circRNAs (DE-circRNAs), a competing endogenous RNA network was constructed, and the expression levels of some DE-circRNAs were verified using quantitative real-time polymerase chain reactions (qRT-PCR).

**Results:**

There were 190 DE-circRNAs identified in the STC group. Bioinformatics analysis predicted that the DE-circRNAs were enriched in the relaxation of smooth muscle, actin binding, actin cytoskeleton organization, dilated cardiomyopathy, and cardiac muscle contraction. These results suggest that muscle diseases may be related to the pathogenesis of STC. The expression levels of the 12 most differentially expressed circRNAs were verified using qRT-PCR. In addition, circRNA–microRNA–mRNA regulatory networks were constructed using the 8 most significant circRNAs. Some mRNAs predicted to be closely related to smooth muscle function were found in these networks.

**Conclusions:**

This study provides a helpful blueprint for researchers to select candidate circRNAs for further study of the pathogenesis of STC and screen potential biomarkers or targets for use in the diagnosis and treatment of STC.

## 1. Introduction

Functional constipation (FC) is a common digestive tract disorder [1]. A recent study showed that the incidence of FC was 8.73% in the Japanese population between the ages of 20 and 69 years [2]. Slow transit constipation (STC) is a typical type of functional constipation characterized by prolonged colonic transmission and a decreased frequency of defecation [3]. Patients with STC suffer from both physical and psychological burdens that seriously affect their quality of life.

Many hypotheses about the pathophysiology of STC have been proposed, including degenerative neuromuscular processes, interstitial cell dysfunction, dysbacteriosis, and autoimmune disorder [4–6]. However, the pathogenesis of STC remains unclear. Recently, the changes and functions in noncoding RNAs (ncRNAs) in the colon tissue of STC patients have been explored, enriching the research directions on the pathogenesis of STC [7–10].

Circular RNAs (circRNAs) are a special class of ncRNAs that have a stable circular structure [11]. These RNAs play important roles in a variety of digestive diseases (e.g., Hirschsprung's disease and colitis) and physiological processes (e.g., the self-renewal of intestinal stem cells) [12–14]. However, the expression profiles of circRNAs in human STC tissues have yet to be explored.

In the present study, we performed whole-transcriptome sequencing of RNA samples from the colon tissues of STC patients and controls to identify differentially expressed circRNAs. Furthermore, the expression of potentially functional circRNAs was validated using reverse transcription quantitative real-time polymerase chain reactions (qRT-PCR). Bioinformatics analyses were also performed to explore the possible regulatory mechanisms of selected circRNAs. This study provides a basis for further research on the pathogenesis of STC, innovative diagnostic methods for STC, and new insights into STC gene therapy.

## 2. Materials and Methods

### 2.1. Tissue Samples and Cell Culture

There were 42 patients included in this study: 21 patients with STC undergoing subtotal colectomy and 21 controls undergoing radical surgery for colon cancer. The inclusion and exclusion criteria were based on those of our previous study [15]. Briefly, all included patients had a history of STC for more than five years, failed to respond to nonsurgical regimens, and had a strong desire for surgery. Patients with obstructed defecation syndrome, severe psychiatric disease, small bowel dysmotility, or megacolon/megarectum were excluded from this study. Tissue samples of these patients were collected during surgical treatment at the Department of Colorectal and Anal Surgery, Zhongnan Hospital of Wuhan University. All samples were obtained from the same region of the colon descendens in the STC and tumor-free control groups. Tissues were immediately frozen in liquid nitrogen for 15 min and stored at −80°C until use. This study was approved by the Ethics Committee of Zhongnan Hospital (ethical application ref: 2022061), and written informed consent was obtained from each participant.

Human HEK 293T cells were purchased from the China Center for Type Culture Collection and cultured in DMEM (Dulbecco's modified Eagle's medium) with 10% fetal bovine serum (Gibco, USA), 1% penicillin/streptomycin, in a humidified incubator with 5% CO_2_ at 37°C.

### 2.2. circRNA Sequencing and Identification

We randomly selected 12 tissues (6 STC and 6 controls) from 42 samples for circRNA screening by Shanghai Majorbio Bio-Pharm Biotechnology Co., Ltd. (Shanghai, China). The remaining 15 pairs of samples were used to verify circRNA expression (Table 1). The sequencing library was prepared using the TruSeq total RNA kit (Illumina; San Diego, CA, USA). First, ribosomal RNA was depleted and fragmented. Next, cDNA was synthesized using random hexamer primers. The RNA template was then removed, and a replacement strand was synthesized incorporating dUTP instead of dTTP to generate double-stranded (ds) cDNA. AMPure XP beads were used to separate the ds-cDNA from the second-strand reaction mix. A single ‘A' nucleotide was added to the 3′ ends of these blunt fragments. Finally, multiple indexing adapters were ligated to the ends of the ds-cDNA. Libraries were size-selected for cDNA target fragments of 200–300 base pairs on 2% Low Range Ultra Agarose, followed by PCR amplification using Phusion DNA polymerase (NEB). After quantification using TBS380, the library was sequenced using an Illumina HiSeq Xten system (Illumina; San Diego, CA, USA). CircRNA Identifier (CIRI) tools were used to identify the circRNAs. The level of each circRNA was calculated using the *spliced reads per billion mapping* (SRPBM) method. Significantly differentially expressed circRNAs (DE-circRNAs) were defined as |log_2_*FC*| > 1 and *p* < 0.001.

### 2.3. Quantitative Real-Time PCR (qRT-PCR)

Total RNA was extracted from tissues using TRIzol reagent (Invitrogen; USA). RNA (1 *μ*g) was reverse-transcribed into cDNA using HiScript II Reverse Transcriptase (Vazyme Biotech Co., Ltd., China). An Applied Biosystems 7500 Real-Time PCR System (ThermoFisher Scientific; USA) was used to perform qRT-PCR in a 10 *μ*l reaction, including 5 *μ*l SYBR Mix (Vazyme Biotech Co., Ltd., China), 1 *μ*l cDNA, 3.6 *μ*l ddH_2_O, and 0.2 *μ*l each of the forward and reverse primers. The qRT-PCR amplification conditions included an initial denaturation step (95°C for 2 min) followed by 40 cycles of denaturation at 95°C for 15 s and annealing at 60°C for 1 min. GAPDH was used as a normalization standard. The relative RNA levels were calculated using the 2^−*ΔΔ*Ct^ method. The experiments were repeated three times for each sample. The primers are listed in Supplementary Table S1.

### 2.4. Functional Enrichment Analysis of Host Genes of the circRNAs

The host genes of the significant DE-circRNAs were subjected to functional enrichment analysis using the Gene Ontology (GO) and Kyoto Encyclopedia of Genes and Genomes (KEGG) databases. The analyses of GO terms and KEGG pathways were performed using Goatools (https://github.com/tanghaibao/Goatools) and KOBAS (http://kobas.cbi.pku.edu.cn/home.do).

### 2.5. Construction of circRNA–microRNA–mRNA Network

The target microRNAs (miRNAs) of the circRNAs were predicted using starBase (https://starbase.sysu.edu.cn/) and circBank (http://www.circbank.cn/index.html). The target mRNAs of the miRNAs were predicted using TargetScan (https://www.targetscan.org/), miRDB (http://mirdb.org/), miRWalk (http://mirwalk.umm.uni-heidelberg.de/), and TarBase (http://microrna.gr/tarbase/). Next, circRNA–miRNA–mRNA interaction networks were constructed and displayed using Cytoscape v3.8.2 [16].

### 2.6. Statistical Analysis

GraphPad Prism 8 was used for data analysis. Two-tailed Student's *t*-test was used to evaluate the differences between the two groups. Data are presented as the mean ± standard deviation. Statistical significance was defined as *p* < 0.05.

## 3. Results

### 3.1. circRNA Expression Profiling in STC

**Figure 1:**
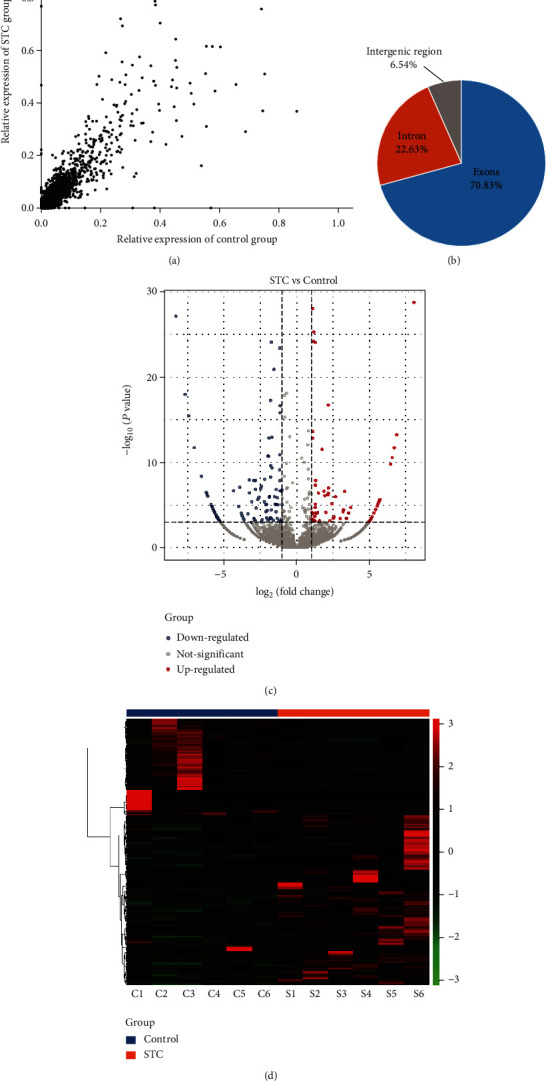
Analysis of the circRNA expression profile in STC. (a) Scatter plot of circRNA expression in the STC and control groups. (b) The circRNAs are classified by distribution. (c) A volcano plot represents the STC DE-circRNAs. Red dots and blue dots indicate the upregulated and downregulated DE-circRNAs, respectively. (d) Hierarchically clustered heatmap analysis of the circRNA expression profiles.

CIRI analysis of the high-throughput RNA sequencing results from the STC and control colon tissues identified 31082 circRNAs (Figure 1(a)). Among the circRNAs, 70.83% was derived from exons, 22.63% were from introns, and 6.54% were intergenic (Figure 1(b)). There were 190 DE-circRNAs; 117 were upregulated and 73 were downregulated.  Figure 1(c) shows a volcano plot of the DE-circRNAs. A hierarchically clustered heatmap revealed distinct differences between the circRNA expression profiles of the two groups (Figure 1(d)). The DE-circRNAs are listed in Supplementary Table S2.

### 3.2. Functional Enrichment Analysis of Host Genes

**Figure 2:**
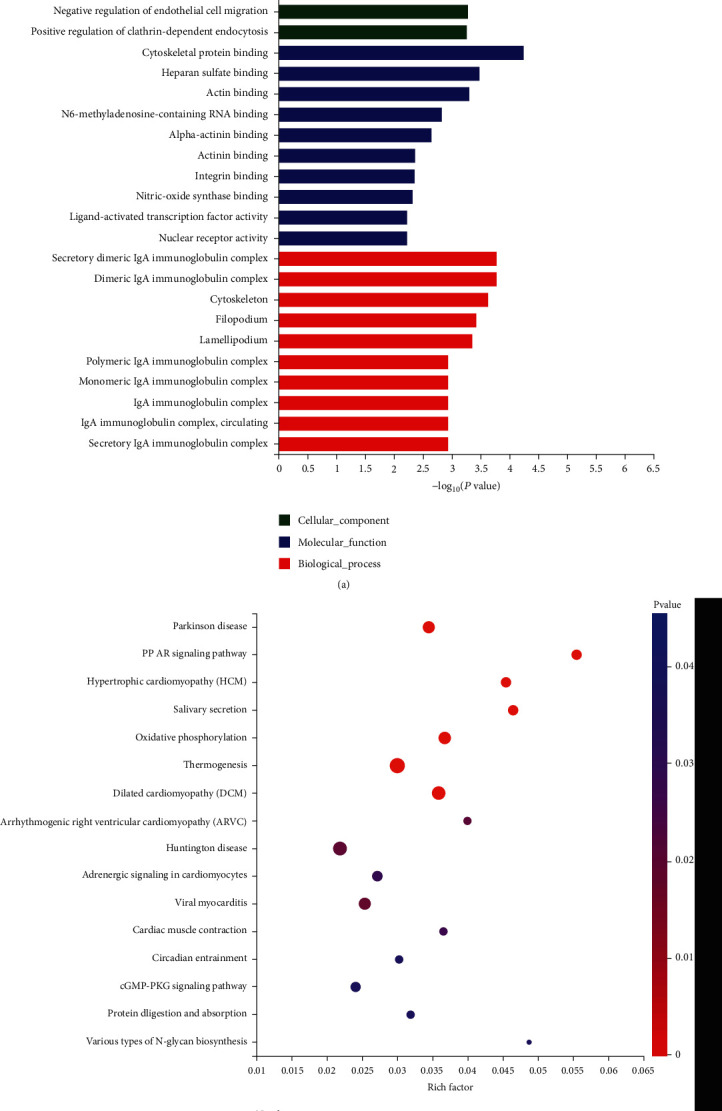
Functional enrichment analysis. (a) Top 10 GO enrichment terms in biological processes (BP), molecular functions (MF), and cellular component (CC) functions. (b) KEGG signaling pathways with *p* < 0.05.

GO enrichment analysis of the host genes of the DE-circRNA transcripts revealed that they were enriched in 555 terms, comprising 413 biological processes (BP) terms, 64 cellular component (CC) terms, and 78 molecular function (MF) terms. The majority of BP host genes were enriched in actin filament–based processes (GO:0030029), the relaxation of smooth muscle (GO:0044557), cytoskeleton organization (GO:0007010), and actin cytoskeletal organization (GO:0030036). The most enriched MF terms were for cytoskeletal protein binding (GO:0008092), heparin sulfate binding (GO:1904399), and actin binding (GO:0003779). Among the CC terms, the host genes were associated primarily with secretory dimeric IgA immunoglobulin complexes (GO:0071752), dimeric IgA immunoglobulin complexes (GO:0071750), and the cytoskeleton (GO:0005856) (Figure 2(a)). In the KEGG signaling pathway analysis, the host genes were significantly enriched in 16 terms; dilated cardiomyopathy (map05414), thermogenesis (map04714), the PPAR signaling pathway (map03320), and oxidative phosphorylation (map00190) were the most significant (Figure 2(b)). The detailed data are provided in Supplementary Tables S3 and S4.

### 3.3. Validation of DE-circRNAs

**Figure 3:**
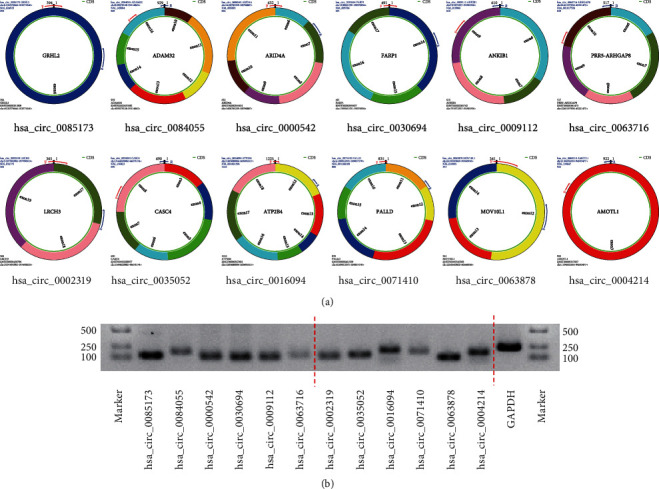
Presence of the selected circRNAs. (a) Detailed information on the circRNAs in circPrimer 2.0. (b) The circRNAs are amplified from cDNA of HEK 293T cells using divergent primers.

**Figure 4:**
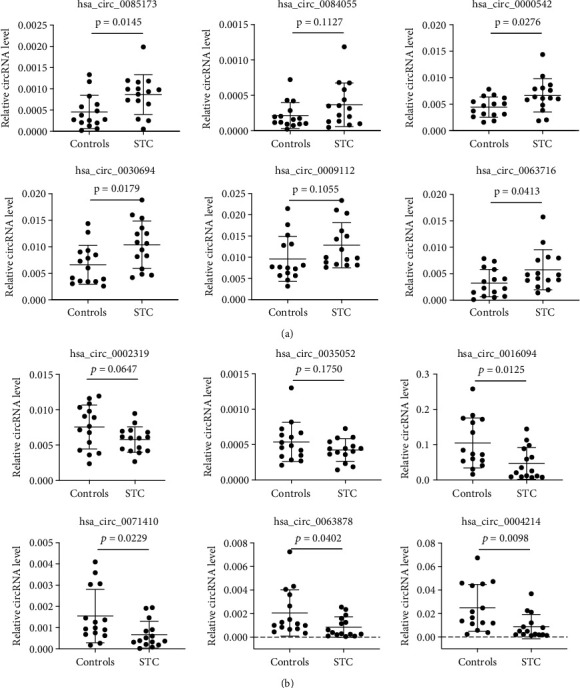
Validation of the expression of the selected circRNAs in STC tissues. (a, b) qRT-PCR analysis of the top 6 up-/downregulated circRNAs in the STC and control groups (*n* = 15 each). All data are means ± SD.

Further analysis of the DE-circRNAs using circBase identified 61 overlapping circRNAs and 129 novel circRNAs [17]. The top 6 DE-circRNAs that were derived from exons were selected for subsequent experiments (Figure 3(a), Table 2). First, divergent primers were designed to amplify the circRNAs from cDNA, and their presence was confirmed using agarose gel electrophoresis (Figure 3(b)). The expression of these DE-circRNAs was verified in 15 pairs of STC and non-STC tissues using qRT-PCR (15 vs. 15 samples). The results revealed that the candidate circRNAs were differentially expressed in the STC samples, which is consistent with the circRNA sequencing results. In the STC tissues, the levels of hsa_circ_0085173, hsa_circ_000542, hsa_circ_0030694, and hsa_circ_0063716 were significantly increased, whereas the levels of hsa_circ_0016094, hsa_circ_0071410, hsa_circ_0063878, and hsa_circ_0004214 were significantly decreased, compared with the control samples (Figures 4(a) and 4(b)).

### 3.4. Construction of the circRNA–miRNA–mRNA Interaction Network in STC

**Figure 5:**
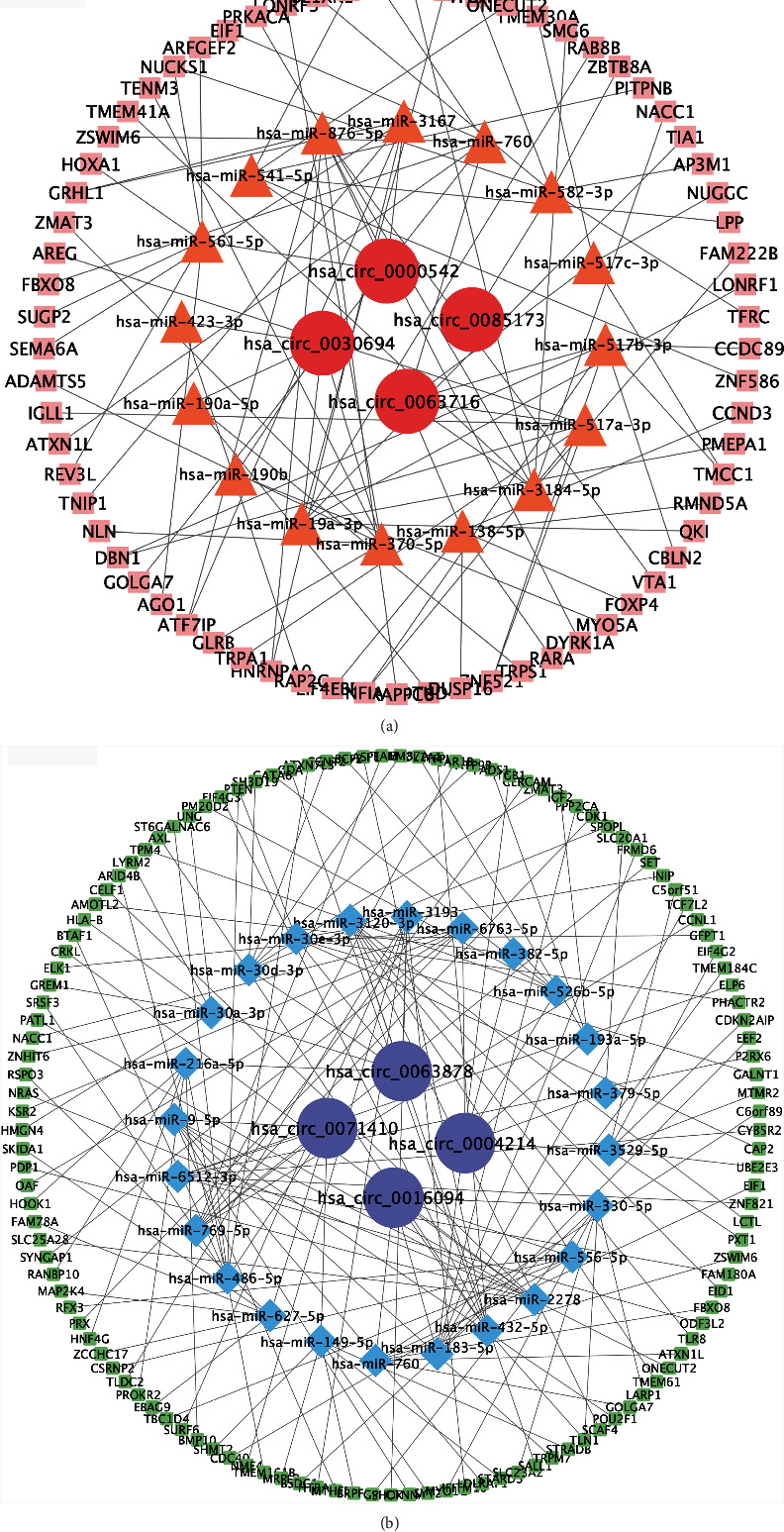
The ceRNA regulatory network. (a) The upregulated ceRNA network. (b) The downregulated ceRNA network.

circRNAs can act as miRNA sponges to regulate miRNA activities and influence downstream mRNA expression. Thus, we constructed a circRNA–miRNA–mRNA interaction network to investigate the role of circRNAs in STC. The starBase and circBank databases were used to predict the target miRNAs of the selected circRNAs. TargetScan, miRDB, miRWalk, and TarBase were used to predict the downstream genes of the identified miRNAs. Because thousands of interaction pairs were predicted for the DE-circRNAs, we established and visualized competing endogenous RNA (ceRNA) networks for the validated circRNAs. Using the regulatory network map, we identified the top 6 miRNAs that potentially bind to the circRNAs and the 6 most likely target genes for each miRNA (Figures 5(a) and 5(b), Supplementary Table S5). This might provide a foundation for understanding the biological functions of circRNAs in STC.

## 4. Discussion

STC is a primary functional disease characterized by impaired colonic function and decreased motility. Surgery may be the definitive therapy for patients with refractory STC who fail to respond to medical treatment [18]. To date, the detailed mechanisms of STC have not been fully elucidated. In recent years, ncRNAs have been confirmed to participate in the occurrence and development of various diseases by directly or indirectly regulating gene expression [19]. Exploring the changes in ncRNAs in STC patients will help enrich our understanding of the pathogenesis and potential therapeutic targets of STC. Using human specimens, we compared circRNA expression patterns between STC and control colon tissues via whole-transcriptome sequencing.

This study identified 190 circRNAs as being significantly differentially expressed in patients with STC compared with controls. GO and KEGG enrichment analyses showed that the host genes of these circRNAs were enriched in the relaxation of smooth muscle, actin binding, dilated cardiomyopathy, and cardiac muscle contraction. These results suggest that STC pathogenesis may be related to myopathy. Smooth muscle cells are the final effectors of gastrointestinal motility [20]. Studies have shown that colonic smooth muscle cells are impaired in patients with STC [21]. Our previous study demonstrated thinning of the intestinal smooth muscle layer in chronically constipated mice [22]. However, whether injury of smooth muscle cells is a primary pathological process of STC or a secondary result of fecal deposition of denervation remains to be further studied. The results of the present study suggest that abnormal expression of circRNAs may be involved in the regulation of smooth muscle injury in patients with STC.

The 12 circRNAs with the most significant abnormal expression were verified via qRT-PCR using 15 paired colon tissues from STC and controls. The results were consistent with the sequencing data. Among the identified circRNAs, four were significantly overexpressed in STC tissues, and four others were significantly downregulated compared with the controls. Since ceRNA mechanisms are important pathophysiological pathways of ncRNAs and ceRNA networks that are out of balance can disrupt life activities and cause disease [23], we constructed circRNA–miRNA–mRNA interaction networks for the top 8 circRNAs that were differentially expressed in both the whole-transcriptome sequencing and qRT-PCR tests.

Interestingly, some mRNAs predicted to be closely related to smooth muscle function were found in these networks. For example, it has been suggested that there may be a ceRNA relationship between hsa_circ_0004214, hsa-miR-526b-5p, and tropomyosin 4 (*TPM4*). TPM4 is a major F-actin–binding protein that plays important roles in modulating muscle contraction [24]. Common binding sites were predicted among hsa_circ_0071410, hsa-miR-149-5p, and synaptotagmin 2 (*SYT2*), thus constituting a potential ceRNA network. SYT2 is a key protein in the neuromuscular junction and is essential for fast synaptic vesicle exocytosis. Studies have shown that *SYT2* is one of the disease genes responsible for congenital myasthenic syndromes [25]. Neuromuscular junction disorders may also play crucial roles in STC; this provides ideas for further understanding of the pathogenesis of the disease. Moreover, hsa_circ_0004214 and hsa-miR-193a-5p shared binding sites with COL1A1, which is the major component of type 1 collagen, suggesting that they may participate in the pathogenesis of STC through ceRNA mechanisms. Although collagen is not a direct component of smooth muscle cell contraction elements, the extracellular matrix composed of collagen plays an important role in regulating muscle contraction [26]. Smooth muscle has been shown to mediate extracellular matrix remodeling, which indirectly regulates overall muscle tissue contractility [27].

Although the ability to bind to miRNAs is the best described mechanism of circRNAs, other functions of circRNAs should not be ignored, including participation as RNA-binding proteins [28], transcriptional regulators [29], and the ability to directly translate proteins [30]. Comprehensive exploration and innovative research on the differentially expressed circRNAs identified in the current study may contribute to further understanding the pathogenesis of STC and provide potential targets for its treatment.

## 5. Conclusions

This is the first study to summarize the differential expression of circRNAs in human STC colon tissues. Bioinformatics methods were used to identify the GO and KEGG pathways of these DE-circRNAs to understand their potential mechanisms of action. Moreover, 12 circRNAs with the most differentially expressed genes were verified using qRT-PCR, and their predicted ceRNA networks were constructed. Taken together, the findings of this study provide a helpful blueprint for researchers to select circRNAs for further study of their corresponding mechanisms. Moreover, it is hoped that our findings will highlight the role of circRNAs in STC and stimulate the exploration and development of new therapeutic targets.

## Figures and Tables

**Table 1:** Demographic information of STC and control.

Characteristics	STC for circRNA screening (*n* = 6)	Control for circRNA screening (*n* = 6)	STC for verification (*n* = 15)	Control for verification (*n* = 15)
Age (y)	62.3 ± 7.0	60.8 ± 10.1	57.3 ± 8.4	60.7 ± 10.6
Male/female	2/4	2/4	4/11	5/10

STC: slow transit constipation.

**Table 2:** Top six up-/downregulated circRNAs.

circRNA ID	circBase ID	Log2FC	*P* value	Regulate
8_101558419_101558812	hsa_circ_0085173	5.80	1.44192*E* − 05	Up
8_39221610_39257343	hsa_circ_0084055	5.60	7.49084*E* − 05	Up
14_58318542_58330169	hsa_circ_0000542	5.52	0.000132928	Up
13_98409338_98424650	hsa_circ_0030694	5.45	0.000237855	Up
7_92343024_92352642	hsa_circ_0009112	5.36	0.000400085	Up
22_44802077_44825593	hsa_circ_0063716	5.28	0.000688839	Up
3_197866112_197871462	hsa_circ_0002319	-5.42	3.5989*E* − 05	Down
15_44328685_44380976	hsa_circ_0035052	-5.12	0.000370208	Down
1_203710877_203722689	hsa_circ_0016094	-5.12	0.000370208	Down
4_168890922_168916027	hsa_circ_0071410	-3.58	8.67005*E* − 05	Down
22_50125392_50128507	hsa_circ_0063878	-3.58	8.67005*E* − 05	Down
11_94799390_94800311	hsa_circ_0004214	-2.67	8.83338*E* − 07	Down

## Data Availability

The authors declare that the data supporting the findings of this study are available within the article and its Supplementary Information File, or from the corresponding authors upon request. Raw data is available at BioSample, accession number PRJNA826640.

## Supplementary Materials

Supplementary 1 Table S1: PCR primers.

Supplementary 2 Table S2: differentially expressed circRNAs in STC.

Supplementary 3 Table S3: the GO enrichment terms.

Supplementary 4 Table S4: the KEGG pathway enrichment terms.

Supplementary 5 Table S5: top 6 upregulated/downregulated circRNA–miRNA–mRNA regulatory network.
