# Supplementary material for: Characterization of Circular RNA Expression Profiles in Colon Specimens of Patients with Slow Transit Constipation
Source: Dis Markers. 2022 Jun 10;2022:3653363. doi: 10.1155/2022/3653363 (PMC9206760; doi:10.1155/2022/3653363)
Supplement: Supplementary 1 — Table S1: PCR primers. [file 3653363.f1.docx]

| Table S1. PCR primers | | | | | | | |
| --- | --- | --- | --- | --- | --- | --- | --- |
| **CircRNA** | **Primer sequence** | **CircRNA** | **Primer sequence** |  |  |  |  |
| hsa_circ_0085173 | F: CAGCCACAGAGAAACTGCCTT | hsa_circ_0002319 | F: AGCTGTATTCATCATTCCCCTG |  |  |  |  |
|  | R: CTCCAGGTGATCTTGATTTAGGG |  | R: ACCTTTGTTGGTTTCTGCCCT |  |  |  |  |
| hsa_circ_0084055 | F: CTAGGTTTCCTCATTGCTCTTCC | hsa_circ_0035052 | F: CTTCAAAACAGAATCCTTCCAGTC |  |  |  |  |
|  | R: AGGTCCACATTGTCGCTACTTG |  | R: ATAAATTGAAGCAAAAGCAACTTG |  |  |  |  |
| hsa_circ_0000542 | F: AAGAAGAGGTGTGTTTGATGATGG | hsa_circ_0016094 | F: CTGCCAGGTGTAATCGAATCC |  |  |  |  |
|  | R: TGGATTTGTTAATGGAAGCTGGT |  | R: ATACAGGTCAGTTCGGTGAGGAT |  |  |  |  |
| hsa_circ_0030694 | F: GGCATGAAGGCTCCCTGACT | hsa_circ_0071410 | F: GCAGAATGGACTGCAAAAGGA |  |  |  |  |
|  | R: TGGTGTCGGGGCTCAGGTT |  | R: CGTATGTTAACCTCGGCTGGC |  |  |  |  |
| hsa_circ_0009112 | F: CTAACGAAACAAGGGTCAAATACA | hsa_circ_0063878 | F: TAGCTTCTGAACATGTCAAATTACAA |  |  |  |  |
|  | R: TCCCAGTCAGCAGAAGAGGTG |  | R: GATCCCGCTCATGTTATACTCTTTC |  |  |  |  |
| hsa_circ_0063716 | F: CCGAAGTTTTGCGGTACGA | hsa_circ_0004214 | F: CAGCCTGTGAGAACAGATGTGGCC |  |  |  |  |
|  | R: CATCCCTATTGCAGACTGACG |  | R: GGTTGGGGTGCCATACCGCAGTTG |  |  |  |  |
| **Gene name** | **Primer sequence** | | |  |  |  |  |
| GAPDH | F: GGAGTCCACTGGCGTCTTCA | R: GTCATGAGTCCTTCCACGATACC | |  |  |  |  |
