# Supplementary material for: Characterization of Circular RNA Expression Profiles in Colon Specimens of Patients with Slow Transit Constipation
Source: Dis Markers. 2022 Jun 10;2022:3653363. doi: 10.1155/2022/3653363 (PMC9206760; doi:10.1155/2022/3653363)
Supplement: Supplementary 2 — Table S2: differentially expressed circRNAs in STC. [file 3653363.f2.docx]

| **Table S2. Differentially expressed circRNAs in STC** | | | | |
| --- | --- | --- | --- | --- |
| **circRNA id** | **Isoform** | **Log2FC(STC_group/Control_group)** | **P value** | **Regulate** |
| 14_106593081_106675318 | intergenic_region | 10.26162052 | 9.40327E-87 | up |
| 2_90082996_90159914 | intergenic_region | 9.602208068 | 3.18043E-60 | up |
| MT_13978_14124 | intron | 9.368535722 | 7.98352E-53 | up |
| 22_22919535_22921435 | intergenic_region | 8.275778581 | 7.69489E-28 | up |
| 5_179724762_179726759 | intron | 7.633330586 | 9.75157E-19 | up |
| 19_41761969_41807389 | intergenic_region | 7.405061598 | 3.08002E-16 | up |
| 10_122580866_122599097 | exon | 7.003699036 | 1.58541E-12 | up |
| 2_191381462_191396497 | exon | 6.523706095 | 4.40289E-09 | up |
| 6_29888467_29943026 | intron | 6.182669177 | 3.15903E-07 | up |
| 2_188995046_188996496 | exon | 6.133759576 | 5.23272E-07 | up |
| 12_6578846_6581349 | intron | 6.133759576 | 5.23272E-07 | up |
| 1_1637762_1704655 | intergenic_region | 6.083133503 | 9.11367E-07 | up |
| 3_112638731_112638875 | intron | 5.860741082 | 8.32641E-06 | up |
| 8_101558419_101558812 | exon | 5.799340537 | 1.44192E-05 | up |
| 7_38242428_38260230 | intergenic_region | 5.799340537 | 1.44192E-05 | up |
| MT_14004_14149 | intron | 5.799340537 | 1.44192E-05 | up |
| MT_13978_14149 | intron | 5.7352102 | 2.54818E-05 | up |
| 15_59671183_59672739 | exon | 5.668096004 | 4.43123E-05 | up |
| 2_88784598_88805408 | intergenic_region | 5.668096004 | 4.43123E-05 | up |
| 19_8337795_8339167 | intron | 5.597706676 | 7.49084E-05 | up |
| 8_39221610_39257343 | exon | 5.597706676 | 7.49084E-05 | up |
| 10_51153164_51246887 | intron | 5.523706095 | 0.000132928 | up |
| 14_58318542_58330169 | exon | 5.523706095 | 0.000132928 | up |
| 14_106235470_106268912 | intergenic_region | 5.445703583 | 0.000237855 | up |
| 3_124737568_124744203 | intron | 5.445703583 | 0.000237855 | up |
| 13_98409338_98424650 | exon | 5.445703583 | 0.000237855 | up |
| 1_1637081_1704146 | intergenic_region | 5.445703583 | 0.000237855 | up |
| 7_92343024_92352642 | exon | 5.363241423 | 0.000400085 | up |
| 3_12416704_12417154 | exon | 5.363241423 | 0.000400085 | up |
| 4_118620414_118628226 | intergenic_region | 5.275778581 | 0.000688839 | up |
| 22_44802077_44825593 | exon | 5.275778581 | 0.000688839 | up |
| 3_47918719_47921878 | exon | 5.275778581 | 0.000688839 | up |
| 4_151352900_151353391 | intergenic_region | 5.275778581 | 0.000688839 | up |
| 2_88947722_88992704 | intergenic_region | 5.275778581 | 0.000688839 | up |
| MT_1678_7693 | intron | 5.275778581 | 0.000688839 | up |
| 1_113848540_113855049 | exon | 4.320172701 | 2.12489E-07 | up |
| 1_95143891_95173889 | exon | 3.976218299 | 1.44192E-05 | up |
| 1_119755427_119759989 | exon | 3.900269446 | 8.17883E-08 | up |
| 15_94440176_94458246 | exon | 3.799340537 | 8.58397E-05 | up |
| 5_179709873_179716294 | exon | 3.7352102 | 0.000159476 | up |
| 1_64802976_64814840 | exon | 3.668096004 | 0.000290801 | up |
| 3_112109543_112113145 | exon | 3.597706676 | 0.00050513 | up |
| 1_200043774_200120955 | exon | 3.33467227 | 0.000101236 | up |
| 9_33948374_33953474 | exon | 3.245404932 | 1.1973E-08 | up |
| 1_20969474_20981227 | exon | 3.239252705 | 3.72678E-07 | up |
| 4_76738761_76741926 | exon | 3.083133503 | 3.94054E-06 | up |
| 3_149846011_149912083 | exon | 3.083133503 | 0.00098698 | up |
| 4_61934840_61948276 | exon | 3.083133503 | 0.00098698 | up |
| 4_71236576_71255399 | exon | 2.948203923 | 1.3642E-08 | up |
| 16_19463280_19464024 | exon | 2.919634771 | 0.000360428 | up |
| 21_37420299_37428908 | exon | 2.919634771 | 0.000360428 | up |
| 6_29890246_29944616 | intron | 2.860741082 | 0.000604189 | up |
| 6_158573420_158589782 | exon | 2.860741082 | 0.000604189 | up |
| 4_68565572_68655182 | intergenic_region | 2.833774034 | 1.37484E-08 | up |
| 3_12312380_12381491 | exon | 2.830367433 | 1.427E-07 | up |
| 19_19640085_19640571 | exon | 2.778278922 | 2.22093E-05 | up |
| 1_113834910_113855049 | exon | 2.668096004 | 2.22507E-06 | up |
| X_2953143_2958435 | exon | 2.597706676 | 1.21676E-06 | up |
| 2_63856306_63859391 | intron | 2.472175794 | 1.1748E-07 | up |
| 19_40623604_40624082 | exon | 2.445703583 | 0.000916403 | up |
| 2_84870225_84870785 | exon | 2.433319858 | 6.30463E-08 | up |
| 16_8915254_8917156 | exon | 2.375314255 | 0.000508966 | up |
| 11_36393846_36419303 | exon | 2.219195053 | 3.99589E-06 | up |
| 1_109661165_109690564 | intergenic_region | 2.197776069 | 9.74746E-09 | up |
| 17_79077430_79077864 | exon | 2.117080835 | 5.65148E-05 | up |
| 16_56626880_56683230 | intergenic_region | 2.112279849 | 9.05267E-06 | up |
| 14_22909483_22911403 | exon | 2.069327704 | 7.85028E-06 | up |
| 19_11724194_11725327 | exon | 2.064274476 | 0.000291905 | up |
| 17_1050050_1100735 | exon | 2.058312331 | 2.73363E-54 | up |
| 4_39898389_39902460 | exon | 2.023012511 | 4.04934E-05 | up |
| 22_28514757_28519320 | intron | 1.986271964 | 0.000701379 | up |
| 14_106538220_106557248 | intergenic_region | 1.972851448 | 1.95753E-11 | up |
| 2_199348701_199433514 | exon | 1.927855278 | 2.08209E-11 | up |
| 8_94664697_94665196 | exon | 1.904796262 | 1.15502E-13 | up |
| 18_62348168_62349937 | exon | 1.882436153 | 1.1906E-06 | up |
| 9_111786793_111787947 | intron | 1.860741082 | 9.61141E-07 | up |
| MT_13846_13989 | intron | 1.860741082 | 0.000399002 | up |
| 12_121780872_121781192 | exon | 1.799340537 | 0.000269612 | up |
| MT_1678_15646 | intron | 1.793626886 | 4.82287E-18 | up |
| 7_22308339_22318037 | exon | 1.788228582 | 5.4293E-09 | up |
| 15_94356137_94402019 | exon | 1.784792229 | 2.46033E-10 | up |
| 15_94356137_94385525 | exon | 1.742904592 | 0.000338216 | up |
| 2_199368605_199433514 | exon | 1.74195493 | 8.16803E-25 | up |
| MT_8927_9189 | intron | 1.741442154 | 3.68717E-05 | up |
| MT_13436_13570 | intron | 1.703199805 | 9.75979E-14 | up |
| 5_145817894_145826200 | exon | 1.686711682 | 4.73032E-10 | up |
| 12_32598497_32611283 | exon | 1.597706676 | 0.0005746 | up |
| MT_8927_9088 | intron | 1.558887427 | 1.20001E-21 | up |
| 17_20204333_20205912 | exon | 1.544267417 | 4.31466E-46 | up |
| 10_7797047_7802854 | exon | 1.530143392 | 3.99849E-57 | up |
| 1_84865385_84866138 | exon | 1.513938318 | 1.02894E-06 | up |
| 2_88124568_88126316 | intron | 1.480893011 | 7.74774E-06 | up |
| 1_113829592_113834439 | exon | 1.480893011 | 7.74774E-06 | up |
| 5_145764931_145826200 | exon | 1.417134431 | 5.87376E-05 | up |
| 3_195959183_195960086 | intergenic_region | 1.405839122 | 1.46923E-07 | up |
| 10_94245946_94246621 | exon | 1.373075783 | 8.58795E-06 | up |
| 19_39877663_39893527 | exon | 1.361498638 | 5.279E-200 | up |
| X_44524002_44527365 | intron | 1.339908919 | 0.000344562 | up |
| 11_114580132_114583021 | exon | 1.32921784 | 1.34257E-06 | up |
| 4_9641071_9641450 | intergenic_region | 1.319604822 | 1.6701E-39 | up |
| 10_94030683_94032252 | exon | 1.290133874 | 8.84053E-06 | up |
| 19_39885673_39899697 | exon | 1.227100618 | 1.65912E-93 | up |
| 3_31576396_31580096 | exon | 1.217675627 | 0.000729001 | up |
| 14_21230319_21234229 | exon | 1.170936215 | 7.47837E-10 | up |
| 1_247156406_247159813 | intron | 1.167562284 | 3.92746E-24 | up |
| 3_149846011_149921227 | exon | 1.163610841 | 1.42952E-11 | up |
| 7_99493040_99494631 | intron | 1.158421631 | 1.42137E-08 | up |
| X_65051462_65075912 | intergenic_region | 1.136615834 | 2.12566E-07 | up |
| MT_13297_13448 | intron | 1.10976863 | 0.000969608 | up |
| 3_172247533_172251541 | exon | 1.106297691 | 1.31076E-16 | up |
| 11_108176246_108177090 | exon | 1.087016938 | 0.000443046 | up |
| 5_21491321_21497196 | intergenic_region | 1.077651438 | 2.09131E-17 | up |
| 2_233388257_233390483 | exon | 1.056883604 | 2.27286E-07 | up |
| 15_75859878_75873568 | exon | 1.038279268 | 0.000692534 | up |
| 6_158282263_158314268 | intergenic_region | 1.017710428 | 0.000763649 | up |
| 7_22291175_22318037 | exon | 1.012744175 | 8.15501E-06 | up |
| 12_69800209_69801721 | exon | 1.003012637 | 1.61975E-07 | up |
| 19_45263113_45263367 | exon | -1.00752822 | 0 | down |
| 4_150467673_150491035 | exon | -1.024984904 | 1.69502E-70 | down |
| 2_40428473_40430301 | exon | -1.026784189 | 7.309E-05 | down |
| 6_32521905_32581838 | intergenic_region | -1.05734772 | 1.26453E-13 | down |
| 10_29512735_29531288 | exon | -1.083143721 | 1.99721E-14 | down |
| 9_16435555_16437524 | exon | -1.09890118 | 1.02714E-28 | down |
| 8_42906173_42943445 | exon | -1.100088321 | 0.000505019 | down |
| 2_237739232_237760205 | exon | -1.116538842 | 9.35816E-05 | down |
| 4_186706563_186709845 | exon | -1.155271236 | 6.64508E-25 | down |
| 11_92352096_92355404 | exon | -1.16479401 | 0.000719136 | down |
| 14_105707832_105708105 | intron | -1.16745581 | 5.04092E-26 | down |
| 1_77631850_77641655 | exon | -1.191347429 | 0.000604189 | down |
| 16_15714909_15715081 | exon | -1.229456727 | 0.000688839 | down |
| 10_101672915_101676436 | exon | -1.229456727 | 0.000688839 | down |
| 17_42610108_42659552 | intergenic_region | -1.238794592 | 0.000195091 | down |
| 10_32543300_32584304 | exon | -1.250767233 | 1.93051E-05 | down |
| 14_105707832_105708129 | intron | -1.2529157 | 8.87446E-25 | down |
| 14_65561337_65561766 | exon | -1.268541935 | 4.78033E-08 | down |
| 1_149655699_149669310 | intergenic_region | -1.275236588 | 7.84393E-06 | down |
| 15_63056985_63062277 | exon | -1.281438929 | 7.90079E-05 | down |
| 4_150435589_150491035 | exon | -1.293329968 | 1.34318E-08 | down |
| 22_16637040_16638740 | intergenic_region | -1.339880717 | 8.87848E-05 | down |
| 14_90943078_91001159 | exon | -1.450046455 | 7.309E-05 | down |
| 10_95351209_95384285 | exon | -1.501828997 | 3.83623E-07 | down |
| 10_29512735_29532172 | exon | -1.567495915 | 0.000681064 | down |
| 18_13681605_13682105 | exon | -1.567495915 | 0.000681064 | down |
| 6_75634707_75678927 | exon | -1.582202414 | 0.000908606 | down |
| 5_39382618_39388852 | exon | -1.769736078 | 2.56063E-12 | down |
| 6_29829418_29888742 | intergenic_region | -1.797141236 | 2.26293E-74 | down |
| 5_81615473_81650339 | exon | -1.89414642 | 2.019E-07 | down |
| 4_155696756_155722192 | exon | -1.958686672 | 6.37912E-07 | down |
| 10_95351209_95410777 | exon | -2.019677302 | 7.309E-05 | down |
| X_32216916_32310276 | exon | -2.17352882 | 5.16536E-07 | down |
| 5_114404438_114404856 | exon | -2.187877623 | 1.75507E-17 | down |
| 7_95225961_95284330 | exon | -2.193706702 | 3.44953E-07 | down |
| 1_240206799_240294883 | exon | -2.202268716 | 8.17883E-08 | down |
| 2_219420105_219420346 | intron | -2.309183919 | 0.000711078 | down |
| 12_103806322_103815121 | exon | -2.424661137 | 7.74774E-06 | down |
| 10_123780167_123799199 | exon | -2.531576341 | 0.000370442 | down |
| 19_10913877_10916777 | exon | -2.572218325 | 0.000242489 | down |
| 11_94799390_94800311 | exon | -2.671753999 | 8.83338E-07 | down |
| 14_106154038_106235368 | intergenic_region | -2.997239913 | 0.000401582 | down |
| 10_21904522_21929141 | exon | -3.183653037 | 6.27535E-05 | down |
| 15_96334076_96337535 | intron | -3.226721759 | 3.99357E-05 | down |
| 7_127373342_127377329 | exon | -3.309183919 | 2.12058E-07 | down |
| 22_22758904_22792578 | intergenic_region | -3.424661137 | 0.000374703 | down |
| 9_16727797_16758273 | intron | -3.424661137 | 0.000374703 | down |
| 22_50125392_50128507 | exon | -3.582202414 | 8.67005E-05 | down |
| 4_168890922_168916027 | exon | -3.582202414 | 8.67005E-05 | down |
| 7_84129123_84194668 | exon | -3.724221419 | 2.04775E-05 | down |
| 7_137251230_137254974 | exon | -4.972148932 | 0.000912713 | down |
| 6_56101472_56141983 | exon | -5.046149514 | 0.000587139 | down |
| 1_203710877_203722689 | exon | -5.116538842 | 0.000370208 | down |
| 15_44328685_44380976 | exon | -5.116538842 | 0.000370208 | down |
| 3_56666260_56673725 | exon | -5.116538842 | 0.000370208 | down |
| 7_33637760_33642918 | intergenic_region | -5.116538842 | 0.000370208 | down |
| 7_26193575_26196481 | exon | -5.183653037 | 0.000237855 | down |
| 10_73396519_73400857 | exon | -5.309183919 | 8.71002E-05 | down |
| 3_197866112_197871462 | exon | -5.424661137 | 3.5989E-05 | down |
| 14_105708359_105708498 | intron | -5.531576341 | 1.40136E-05 | down |
| 10_27673960_27675554 | intron | -5.582202414 | 8.58795E-06 | down |
| 1_143745217_143760658 | intergenic_region | -5.631112014 | 5.53404E-06 | down |
| 14_106117039_106211242 | intergenic_region | -5.678417729 | 3.42777E-06 | down |
| 14_105707850_105708125 | intron | -5.724221419 | 2.09173E-06 | down |
| 14_106791108_106828163 | intergenic_region | -6.452141873 | 1.4111E-10 | down |
| 14_105707929_105708105 | intron | -6.557111433 | 2.34331E-11 | down |
| MT_1678_11959 | intron | -6.701501342 | 1.63926E-12 | down |
| MT_957_12079 | intron | -6.873968538 | 5.22835E-14 | down |
| 6_32530125_32584378 | intergenic_region | -8.046149514 | 1.92927E-29 | down |
| 19_41718128_41720208 | exon | -8.382432901 | 4.26963E-36 | down |
| 19_41715650_41717733 | exon | -9.407216002 | 1.81285E-65 | down |
| 6_29887955_29942626 | intergenic_region | -10.10359979 | 2.15892E-96 | down |
| 6_31271073_31355592 | intergenic_region | -12.88188415 | 0 | down |
