# Supplementary material for: Characterization of Circular RNA Expression Profiles in Colon Specimens of Patients with Slow Transit Constipation
Source: Dis Markers. 2022 Jun 10;2022:3653363. doi: 10.1155/2022/3653363 (PMC9206760; doi:10.1155/2022/3653363)
Supplement: Supplementary 3 — Table S3: the GO enrichment terms. [file 3653363.f3.docx]

| **Table S3. The GO enrichment terms.** | | | | | | |
| --- | --- | --- | --- | --- | --- | --- |
| **Number** | **GO id** | **Term type** | **Description** | **Ratio_in_pop** | **pvalue** |  |
| 15 | GO:0030029 | BP | actin filament-based process | 431/18359 | 8.09762E-07 |  |
| 3 | GO:0044557 | BP | relaxation of smooth muscle | 7/18359 | 1.39204E-05 |  |
| 20 | GO:0007010 | BP | cytoskeleton organization | 942/18359 | 2.26478E-05 |  |
| 20 | GO:0008092 | MF | cytoskeletal protein binding | 1005/18359 | 5.63375E-05 |  |
| 11 | GO:0030036 | BP | actin cytoskeleton organization | 376/18359 | 0.000121528 |  |
| 5 | GO:0010633 | BP | negative regulation of epithelial cell migration | 67/18359 | 0.000143284 |  |
| 12 | GO:0097435 | BP | supramolecular fiber organization | 459/18359 | 0.000170801 |  |
| 2 | GO:0071752 | CC | secretory dimeric IgA immunoglobulin complex | 3/18359 | 0.000165033 |  |
| 2 | GO:0071750 | CC | dimeric IgA immunoglobulin complex | 3/18359 | 0.000165033 |  |
| 25 | GO:0005856 | CC | cytoskeleton | 1521/18359 | 0.000231014 |  |
| 10 | GO:0019932 | BP | second-messenger-mediated signaling | 345/18359 | 0.000268007 |  |
| 3 | GO:0090075 | BP | relaxation of muscle | 18/18359 | 0.00030558 |  |
| 2 | GO:1904399 | MF | heparan sulfate binding | 4/18359 | 0.00032845 |  |
| 5 | GO:0030175 | CC | filopodium | 82/18359 | 0.00037022 |  |
| 7 | GO:0030027 | CC | lamellipodium | 182/18359 | 0.000436682 |  |
| 4 | GO:0010596 | BP | negative regulation of endothelial cell migration | 50/18359 | 0.000523754 |  |
| 2 | GO:2000370 | BP | positive regulation of clathrin-dependent endocytosis | 5/18359 | 0.000544735 |  |
| 11 | GO:0003779 | MF | actin binding | 444/18359 | 0.00050416 |  |
| 8 | GO:0003012 | BP | muscle system process | 257/18359 | 0.000699441 |  |
| 8 | GO:0030336 | BP | negative regulation of cell migration | 260/18359 | 0.000754311 |  |
| 9 | GO:0070482 | BP | response to oxygen levels | 325/18359 | 0.00075443 |  |
| 8 | GO:0007015 | BP | actin filament organization | 261/18359 | 0.000773353 |  |
| 2 | GO:0015990 | BP | electron transport coupled proton transport | 6/18359 | 0.000813103 |  |
| 2 | GO:0060087 | BP | relaxation of vascular smooth muscle | 6/18359 | 0.000813103 |  |
| 2 | GO:1902083 | BP | negative regulation of peptidyl-cysteine S-nitrosylation | 6/18359 | 0.000813103 |  |
| 2 | GO:0015988 | BP | energy coupled proton transmembrane transport, against electrochemical gradient | 6/18359 | 0.000813103 |  |
| 6 | GO:0019722 | BP | calcium-mediated signaling | 149/18359 | 0.000887553 |  |
| 8 | GO:2000146 | BP | negative regulation of cell motility | 275/18359 | 0.00108306 |  |
| 4 | GO:0051017 | BP | actin filament bundle assembly | 61/18359 | 0.001114046 |  |
| 2 | GO:0071749 | CC | polymeric IgA immunoglobulin complex | 7/18359 | 0.001132777 |  |
| 2 | GO:0071748 | CC | monomeric IgA immunoglobulin complex | 7/18359 | 0.001132777 |  |
| 2 | GO:0071745 | CC | IgA immunoglobulin complex | 7/18359 | 0.001132777 |  |
| 2 | GO:0071746 | CC | IgA immunoglobulin complex, circulating | 7/18359 | 0.001132777 |  |
| 2 | GO:0071751 | CC | secretory IgA immunoglobulin complex | 7/18359 | 0.001132777 |  |
| 9 | GO:0051056 | BP | regulation of small GTPase mediated signal transduction | 350/18359 | 0.001267742 |  |
| 4 | GO:0061572 | BP | actin filament bundle organization | 64/18359 | 0.001333219 |  |
| 6 | GO:0010594 | BP | regulation of endothelial cell migration | 162/18359 | 0.001366474 |  |
| 55 | GO:0032502 | BP | developmental process | 5047/18359 | 0.001421558 |  |
| 7 | GO:0010632 | BP | regulation of epithelial cell migration | 224/18359 | 0.001475784 |  |
| 2 | GO:0060267 | BP | positive regulation of respiratory burst | 8/18359 | 0.001502985 |  |
| 2 | GO:0010749 | BP | regulation of nitric oxide mediated signal transduction | 8/18359 | 0.001502985 |  |
| 2 | GO:0140059 | BP | dendrite arborization | 8/18359 | 0.001502985 |  |
| 6 | GO:0098858 | CC | actin-based cell projection | 164/18359 | 0.001454874 |  |
| 6 | GO:0043197 | CC | dendritic spine | 164/18359 | 0.001454874 |  |
| 2 | GO:1990247 | MF | N6-methyladenosine-containing RNA binding | 8/18359 | 0.001502985 |  |
| 6 | GO:0044309 | CC | neuron spine | 166/18359 | 0.001547571 |  |
| 20 | GO:0007275 | BP | multicellular organism development | 1269/18359 | 0.001768289 |  |
| 5 | GO:0001764 | BP | neuron migration | 117/18359 | 0.001847084 |  |
| 58 | GO:0016043 | BP | cellular component organization | 5469/18359 | 0.001866892 |  |
| 2 | GO:2000169 | BP | regulation of peptidyl-cysteine S-nitrosylation | 9/18359 | 0.001922968 |  |
| 2 | GO:1901660 | BP | calcium ion export | 9/18359 | 0.001922968 |  |
| 2 | GO:0098917 | BP | retrograde trans-synaptic signaling | 9/18359 | 0.001922968 |  |
| 2 | GO:0098974 | BP | postsynaptic actin cytoskeleton organization | 9/18359 | 0.001922968 |  |
| 7 | GO:0044449 | CC | contractile fiber part | 230/18359 | 0.001715925 |  |
| 8 | GO:0051271 | BP | negative regulation of cellular component movement | 309/18359 | 0.002255463 |  |
| 3 | GO:0051393 | MF | alpha-actinin binding | 35/18359 | 0.002234182 |  |
| 2 | GO:0070106 | BP | interleukin-27-mediated signaling pathway | 10/18359 | 0.002391973 |  |
| 2 | GO:0070757 | BP | interleukin-35-mediated signaling pathway | 10/18359 | 0.002391973 |  |
| 3 | GO:0003382 | BP | epithelial cell morphogenesis | 36/18359 | 0.002424087 |  |
| 58 | GO:0071840 | BP | cellular component organization or biogenesis | 5548/18359 | 0.002687334 |  |
| 7 | GO:0044455 | CC | mitochondrial membrane part | 249/18359 | 0.002681483 |  |
| 5 | GO:0030018 | CC | Z disc | 128/18359 | 0.002732964 |  |
| 2 | GO:0099188 | BP | postsynaptic cytoskeleton organization | 11/18359 | 0.002909256 |  |
| 8 | GO:0040013 | BP | negative regulation of locomotion | 324/18359 | 0.003016694 |  |
| 41 | GO:0048856 | BP | anatomical structure development | 3597/18359 | 0.003397214 |  |
| 15 | GO:0030334 | BP | regulation of cell migration | 872/18359 | 0.003435025 |  |
| 2 | GO:1900272 | BP | negative regulation of long-term synaptic potentiation | 12/18359 | 0.00347408 |  |
| 2 | GO:0021604 | BP | cranial nerve structural organization | 12/18359 | 0.00347408 |  |
| 3 | GO:0001937 | BP | negative regulation of endothelial cell proliferation | 42/18359 | 0.003772726 |  |
| 2 | GO:0045986 | BP | negative regulation of smooth muscle contraction | 13/18359 | 0.004085718 |  |
| 2 | GO:0140058 | BP | neuron projection arborization | 13/18359 | 0.004085718 |  |
| 5 | GO:0071453 | BP | cellular response to oxygen levels | 142/18359 | 0.004261606 |  |
| 3 | GO:0048813 | BP | dendrite morphogenesis | 44/18359 | 0.004305533 |  |
| 15 | GO:2000145 | BP | regulation of cell motility | 937/18359 | 0.005053397 |  |
| 6 | GO:0006936 | BP | muscle contraction | 213/18359 | 0.005310049 |  |
| 2 | GO:0036376 | BP | sodium ion export across plasma membrane | 15/18359 | 0.005446564 |  |
| 2 | GO:0046321 | BP | positive regulation of fatty acid oxidation | 15/18359 | 0.005446564 |  |
| 3 | GO:0006119 | BP | oxidative phosphorylation | 48/18359 | 0.0055024 |  |
| 3 | GO:0007173 | BP | epidermal growth factor receptor signaling pathway | 48/18359 | 0.0055024 |  |
| 5 | GO:0043484 | BP | regulation of RNA splicing | 151/18359 | 0.005518986 |  |
| 6 | GO:0090257 | BP | regulation of muscle system process | 219/18359 | 0.006062957 |  |
| 3 | GO:0051972 | BP | regulation of telomerase activity | 50/18359 | 0.006168319 |  |
| 2 | GO:0060263 | BP | regulation of respiratory burst | 16/18359 | 0.006194356 |  |
| 12 | GO:0048468 | BP | cell development | 701/18359 | 0.006283867 |  |
| 5 | GO:0006937 | BP | regulation of muscle contraction | 156/18359 | 0.006320697 |  |
| 2 | GO:0060850 | BP | regulation of transcription involved in cell fate commitment | 17/18359 | 0.006986131 |  |
| 2 | GO:1904706 | BP | negative regulation of vascular smooth muscle cell proliferation | 17/18359 | 0.006986131 |  |
| 2 | GO:0035994 | BP | response to muscle stretch | 17/18359 | 0.006986131 |  |
| 2 | GO:0009415 | BP | response to water | 17/18359 | 0.006986131 |  |
| 7 | GO:0036293 | BP | response to decreased oxygen levels | 299/18359 | 0.007217833 |  |
| 1 | GO:1903045 | BP | neural crest cell migration involved in sympathetic nervous system development | 1/18359 | 0.00746228 |  |
| 1 | GO:0098736 | BP | negative regulation of the force of heart contraction | 1/18359 | 0.00746228 |  |
| 1 | GO:0051758 | BP | homologous chromosome movement towards spindle pole involved in homologous chromosome segregation | 1/18359 | 0.00746228 |  |
| 1 | GO:0033242 | BP | negative regulation of cellular amine catabolic process | 1/18359 | 0.00746228 |  |
| 1 | GO:0033241 | BP | regulation of cellular amine catabolic process | 1/18359 | 0.00746228 |  |
| 1 | GO:1900081 | BP | regulation of arginine catabolic process | 1/18359 | 0.00746228 |  |
| 1 | GO:1900082 | BP | negative regulation of arginine catabolic process | 1/18359 | 0.00746228 |  |
| 1 | GO:1904389 | BP | rod bipolar cell differentiation | 1/18359 | 0.00746228 |  |
| 1 | GO:0009956 | BP | radial pattern formation | 1/18359 | 0.00746228 |  |
| 1 | GO:0045713 | BP | low-density lipoprotein particle receptor biosynthetic process | 1/18359 | 0.00746228 |  |
| 1 | GO:0042669 | BP | regulation of inner ear auditory receptor cell fate specification | 1/18359 | 0.00746228 |  |
| 1 | GO:0043990 | BP | histone H2A-S1 phosphorylation | 1/18359 | 0.00746228 |  |
| 1 | GO:1903249 | BP | negative regulation of citrulline biosynthetic process | 1/18359 | 0.00746228 |  |
| 1 | GO:1903248 | BP | regulation of citrulline biosynthetic process | 1/18359 | 0.00746228 |  |
| 1 | GO:0052551 | BP | response to defense-related nitric oxide production by other organism involved in symbiotic interaction | 1/18359 | 0.00746228 |  |
| 1 | GO:0052565 | BP | response to defense-related host nitric oxide production | 1/18359 | 0.00746228 |  |
| 1 | GO:2000283 | BP | negative regulation of cellular amino acid biosynthetic process | 1/18359 | 0.00746228 |  |
| 1 | GO:1904391 | BP | response to ciliary neurotrophic factor | 1/18359 | 0.00746228 |  |
| 1 | GO:0021629 | BP | olfactory nerve structural organization | 1/18359 | 0.00746228 |  |
| 1 | GO:0098925 | BP | retrograde trans-synaptic signaling by nitric oxide, modulating synaptic transmission | 1/18359 | 0.00746228 |  |
| 1 | GO:0035644 | BP | phosphoanandamide dephosphorylation | 1/18359 | 0.00746228 |  |
| 1 | GO:2000230 | BP | negative regulation of pancreatic stellate cell proliferation | 1/18359 | 0.00746228 |  |
| 3 | GO:0007007 | BP | inner mitochondrial membrane organization | 54/18359 | 0.007639256 |  |
| 3 | GO:0006939 | BP | smooth muscle contraction | 54/18359 | 0.007639256 |  |
| 4 | GO:0043279 | BP | response to alkaloid | 104/18359 | 0.007676564 |  |
| 2 | GO:0048532 | BP | anatomical structure arrangement | 18/18359 | 0.0078212 |  |
| 2 | GO:0010881 | BP | regulation of cardiac muscle contraction by regulation of the release of sequestered calcium ion | 18/18359 | 0.0078212 |  |
| 2 | GO:2000369 | BP | regulation of clathrin-dependent endocytosis | 18/18359 | 0.0078212 |  |
| 2 | GO:0003094 | BP | glomerular filtration | 18/18359 | 0.0078212 |  |
| 2 | GO:0036270 | BP | response to diuretic | 18/18359 | 0.0078212 |  |
| 2 | GO:0031000 | BP | response to caffeine | 18/18359 | 0.0078212 |  |
| 30 | GO:0006996 | BP | organelle organization | 2499/18359 | 0.00803474 |  |
| 3 | GO:0019731 | BP | antibacterial humoral response | 56/18359 | 0.008445724 |  |
| 7 | GO:0030855 | BP | epithelial cell differentiation | 309/18359 | 0.008570299 |  |
| 2 | GO:0097205 | BP | renal filtration | 19/18359 | 0.008698882 |  |
| 2 | GO:0071295 | BP | cellular response to vitamin | 19/18359 | 0.008698882 |  |
| 3 | GO:0006940 | BP | regulation of smooth muscle contraction | 58/18359 | 0.009300385 |  |
| 4 | GO:2000278 | BP | regulation of DNA biosynthetic process | 110/18359 | 0.009315136 |  |
| 4 | GO:0007229 | BP | integrin-mediated signaling pathway | 112/18359 | 0.009908091 |  |
| 22 | GO:0035556 | BP | intracellular signal transduction | 1665/18359 | 0.010020601 |  |
| 3 | GO:0007519 | BP | skeletal muscle tissue development | 61/18359 | 0.01067397 |  |
| 4 | GO:0071456 | BP | cellular response to hypoxia | 116/18359 | 0.011166341 |  |
| 4 | GO:0016525 | BP | negative regulation of angiogenesis | 116/18359 | 0.011166341 |  |
| 2 | GO:0010882 | BP | regulation of cardiac muscle contraction by calcium ion signaling | 22/18359 | 0.011580919 |  |
| 2 | GO:0048025 | BP | negative regulation of mRNA splicing, via spliceosome | 22/18359 | 0.011580919 |  |
| 20 | GO:0006928 | BP | movement of cell or subcellular component | 1526/18359 | 0.012131884 |  |
| 4 | GO:0042542 | BP | response to hydrogen peroxide | 119/18359 | 0.012174675 |  |
| 4 | GO:2000181 | BP | negative regulation of blood vessel morphogenesis | 119/18359 | 0.012174675 |  |
| 15 | GO:0040012 | BP | regulation of locomotion | 1012/18359 | 0.01229808 |  |
| 15 | GO:0051270 | BP | regulation of cellular component movement | 1014/18359 | 0.01239486 |  |
| 2 | GO:0055093 | BP | response to hyperoxia | 23/18359 | 0.012622402 |  |
| 2 | GO:0048714 | BP | positive regulation of oligodendrocyte differentiation | 23/18359 | 0.012622402 |  |
| 2 | GO:0042776 | BP | mitochondrial ATP synthesis coupled proton transport | 23/18359 | 0.012622402 |  |
| 2 | GO:0045932 | BP | negative regulation of muscle contraction | 23/18359 | 0.012622402 |  |
| 2 | GO:0046339 | BP | diacylglycerol metabolic process | 23/18359 | 0.012622402 |  |
| 9 | GO:0032989 | BP | cellular component morphogenesis | 500/18359 | 0.012766726 |  |
| 13 | GO:0010243 | BP | response to organonitrogen compound | 854/18359 | 0.012950575 |  |
| 6 | GO:0007568 | BP | aging | 258/18359 | 0.012967365 |  |
| 3 | GO:0038127 | BP | ERBB signaling pathway | 67/18359 | 0.013756674 |  |
| 4 | GO:0036294 | BP | cellular response to decreased oxygen levels | 125/18359 | 0.014362276 |  |
| 4 | GO:0050680 | BP | negative regulation of epithelial cell proliferation | 126/18359 | 0.014749472 |  |
| 2 | GO:0071168 | BP | protein localization to chromatin | 25/18359 | 0.014822702 |  |
| 2 | GO:0060330 | BP | regulation of response to interferon-gamma | 25/18359 | 0.014822702 |  |
| 2 | GO:0060334 | BP | regulation of interferon-gamma-mediated signaling pathway | 25/18359 | 0.014822702 |  |
| 1 | GO:0060857 | BP | establishment of glial blood-brain barrier | 2/18359 | 0.014869278 |  |
| 1 | GO:0034971 | BP | histone H3-R17 methylation | 2/18359 | 0.014869278 |  |
| 1 | GO:0034970 | BP | histone H3-R2 methylation | 2/18359 | 0.014869278 |  |
| 1 | GO:0099554 | BP | trans-synaptic signaling by soluble gas, modulating synaptic transmission | 2/18359 | 0.014869278 |  |
| 1 | GO:0099555 | BP | trans-synaptic signaling by nitric oxide, modulating synaptic transmission | 2/18359 | 0.014869278 |  |
| 1 | GO:1904049 | BP | negative regulation of spontaneous neurotransmitter secretion | 2/18359 | 0.014869278 |  |
| 1 | GO:0140199 | BP | negative regulation of adenylate cyclase-activating adrenergic receptor signaling pathway involved in heart process | 2/18359 | 0.014869278 |  |
| 1 | GO:0031104 | BP | dendrite regeneration | 2/18359 | 0.014869278 |  |
| 1 | GO:1902073 | BP | positive regulation of hypoxia-inducible factor-1alpha signaling pathway | 2/18359 | 0.014869278 |  |
| 1 | GO:1905145 | BP | cellular response to acetylcholine | 2/18359 | 0.014869278 |  |
| 1 | GO:2000229 | BP | regulation of pancreatic stellate cell proliferation | 2/18359 | 0.014869278 |  |
| 1 | GO:1904808 | BP | positive regulation of protein oxidation | 2/18359 | 0.014869278 |  |
| 1 | GO:1904806 | BP | regulation of protein oxidation | 2/18359 | 0.014869278 |  |
| 1 | GO:1902767 | BP | isoprenoid biosynthetic process via mevalonate | 2/18359 | 0.014869278 |  |
| 1 | GO:1990428 | BP | miRNA transport | 2/18359 | 0.014869278 |  |
| 1 | GO:0060849 | BP | regulation of transcription involved in lymphatic endothelial cell fate commitment | 2/18359 | 0.014869278 |  |
| 1 | GO:0009405 | BP | pathogenesis | 2/18359 | 0.014869278 |  |
| 1 | GO:0071848 | BP | positive regulation of ERK1 and ERK2 cascade via TNFSF11-mediated signaling | 2/18359 | 0.014869278 |  |
| 1 | GO:1905663 | BP | positive regulation of telomerase RNA reverse transcriptase activity | 2/18359 | 0.014869278 |  |
| 1 | GO:0045763 | BP | negative regulation of cellular amino acid metabolic process | 2/18359 | 0.014869278 |  |
| 1 | GO:0071306 | BP | cellular response to vitamin E | 2/18359 | 0.014869278 |  |
| 1 | GO:0006580 | BP | ethanolamine metabolic process | 2/18359 | 0.014869278 |  |
| 1 | GO:0071812 | BP | positive regulation of fever generation by positive regulation of prostaglandin secretion | 2/18359 | 0.014869278 |  |
| 1 | GO:0035026 | BP | leading edge cell differentiation | 2/18359 | 0.014869278 |  |
| 1 | GO:1901558 | BP | response to metformin | 2/18359 | 0.014869278 |  |
| 1 | GO:0045213 | BP | neurotransmitter receptor metabolic process | 2/18359 | 0.014869278 |  |
| 1 | GO:0090132 | BP | epithelium migration | 2/18359 | 0.014869278 |  |
| 1 | GO:1904397 | BP | negative regulation of neuromuscular junction development | 2/18359 | 0.014869278 |  |
| 1 | GO:0036071 | BP | N-glycan fucosylation | 2/18359 | 0.014869278 |  |
| 1 | GO:0098923 | BP | retrograde trans-synaptic signaling by soluble gas | 2/18359 | 0.014869278 |  |
| 1 | GO:0098924 | BP | retrograde trans-synaptic signaling by nitric oxide | 2/18359 | 0.014869278 |  |
| 1 | GO:0033578 | BP | protein glycosylation in Golgi | 2/18359 | 0.014869278 |  |
| 1 | GO:1904373 | BP | response to kainic acid | 2/18359 | 0.014869278 |  |
| 1 | GO:0060694 | BP | regulation of cholesterol transporter activity | 2/18359 | 0.014869278 |  |
| 1 | GO:0097355 | BP | protein localization to heterochromatin | 2/18359 | 0.014869278 |  |
| 1 | GO:0003420 | BP | regulation of growth plate cartilage chondrocyte proliferation | 2/18359 | 0.014869278 |  |
| 1 | GO:0010142 | BP | farnesyl diphosphate biosynthetic process, mevalonate pathway | 2/18359 | 0.014869278 |  |
| 4 | GO:0002064 | BP | epithelial cell development | 128/18359 | 0.01554355 |  |
| 5 | GO:0009612 | BP | response to mechanical stimulus | 195/18359 | 0.015550097 |  |
| 2 | GO:0010880 | BP | regulation of release of sequestered calcium ion into cytosol by sarcoplasmic reticulum | 26/18359 | 0.015980254 |  |
| 2 | GO:0002026 | BP | regulation of the force of heart contraction | 26/18359 | 0.015980254 |  |
| 2 | GO:0033119 | BP | negative regulation of RNA splicing | 26/18359 | 0.015980254 |  |
| 2 | GO:0015985 | BP | energy coupled proton transport, down electrochemical gradient | 26/18359 | 0.015980254 |  |
| 2 | GO:0015986 | BP | ATP synthesis coupled proton transport | 26/18359 | 0.015980254 |  |
| 3 | GO:0032272 | BP | negative regulation of protein polymerization | 71/18359 | 0.016064339 |  |
| 4 | GO:1901343 | BP | negative regulation of vasculature development | 130/18359 | 0.016364096 |  |
| 6 | GO:0006898 | BP | receptor-mediated endocytosis | 272/18359 | 0.016427618 |  |
| 3 | GO:0030048 | BP | actin filament-based movement | 72/18359 | 0.016673141 |  |
| 4 | GO:1902600 | BP | proton transmembrane transport | 131/18359 | 0.016784374 |  |
| 15 | GO:0048870 | BP | cell motility | 1078/18359 | 0.017005659 |  |
| 2 | GO:0035902 | BP | response to immobilization stress | 28/18359 | 0.018407056 |  |
| 9 | GO:0098662 | BP | inorganic cation transmembrane transport | 534/18359 | 0.018793962 |  |
| 14 | GO:0016477 | BP | cell migration | 973/18359 | 0.018859302 |  |
| 6 | GO:0050768 | BP | negative regulation of neurogenesis | 281/18359 | 0.018962011 |  |
| 6 | GO:0001666 | BP | response to hypoxia | 286/18359 | 0.020479817 |  |
| 4 | GO:0062013 | BP | positive regulation of small molecule metabolic process | 140/18359 | 0.020872359 |  |
| 2 | GO:0036296 | BP | response to increased oxygen levels | 30/18359 | 0.020978725 |  |
| 2 | GO:0034110 | BP | regulation of homotypic cell-cell adhesion | 30/18359 | 0.020978725 |  |
| 3 | GO:0002028 | BP | regulation of sodium ion transport | 80/18359 | 0.022005967 |  |
| 1 | GO:0010751 | BP | negative regulation of nitric oxide mediated signal transduction | 3/18359 | 0.0222214 |  |
| 1 | GO:0098942 | BP | retrograde trans-synaptic signaling by trans-synaptic protein complex | 3/18359 | 0.0222214 |  |
| 1 | GO:0021856 | BP | hypothalamic tangential migration using cell-axon interactions | 3/18359 | 0.0222214 |  |
| 1 | GO:0110009 | BP | formin-nucleated actin cable organization | 3/18359 | 0.0222214 |  |
| 1 | GO:1903375 | BP | facioacoustic ganglion development | 3/18359 | 0.0222214 |  |
| 1 | GO:1904048 | BP | regulation of spontaneous neurotransmitter secretion | 3/18359 | 0.0222214 |  |
| 1 | GO:0140192 | BP | regulation of adenylate cyclase-activating adrenergic receptor signaling pathway involved in heart process | 3/18359 | 0.0222214 |  |
| 1 | GO:0070433 | BP | negative regulation of nucleotide-binding oligomerization domain containing 2 signaling pathway | 3/18359 | 0.0222214 |  |
| 1 | GO:0043476 | BP | pigment accumulation | 3/18359 | 0.0222214 |  |
| 1 | GO:1902523 | BP | positive regulation of protein K63-linked ubiquitination | 3/18359 | 0.0222214 |  |
| 1 | GO:0061713 | BP | anterior neural tube closure | 3/18359 | 0.0222214 |  |
| 1 | GO:0071878 | BP | negative regulation of adenylate cyclase-activating adrenergic receptor signaling pathway | 3/18359 | 0.0222214 |  |
| 1 | GO:0070649 | BP | formin-nucleated actin cable assembly | 3/18359 | 0.0222214 |  |
| 1 | GO:0044205 | BP | 'de novo' UMP biosynthetic process | 3/18359 | 0.0222214 |  |
| 1 | GO:0003365 | BP | establishment of cell polarity involved in ameboidal cell migration | 3/18359 | 0.0222214 |  |
| 1 | GO:2000282 | BP | regulation of cellular amino acid biosynthetic process | 3/18359 | 0.0222214 |  |
| 1 | GO:0090234 | BP | regulation of kinetochore assembly | 3/18359 | 0.0222214 |  |
| 1 | GO:0060414 | BP | aorta smooth muscle tissue morphogenesis | 3/18359 | 0.0222214 |  |
| 1 | GO:1905661 | BP | regulation of telomerase RNA reverse transcriptase activity | 3/18359 | 0.0222214 |  |
| 1 | GO:0034157 | BP | positive regulation of toll-like receptor 7 signaling pathway | 3/18359 | 0.0222214 |  |
| 1 | GO:0043482 | BP | cellular pigment accumulation | 3/18359 | 0.0222214 |  |
| 1 | GO:1900039 | BP | positive regulation of cellular response to hypoxia | 3/18359 | 0.0222214 |  |
| 1 | GO:0043686 | BP | co-translational protein modification | 3/18359 | 0.0222214 |  |
| 1 | GO:1902775 | BP | mitochondrial large ribosomal subunit assembly | 3/18359 | 0.0222214 |  |
| 1 | GO:0043988 | BP | histone H3-S28 phosphorylation | 3/18359 | 0.0222214 |  |
| 1 | GO:1904395 | BP | positive regulation of skeletal muscle acetylcholine-gated channel clustering | 3/18359 | 0.0222214 |  |
| 1 | GO:0099548 | BP | trans-synaptic signaling by nitric oxide | 3/18359 | 0.0222214 |  |
| 1 | GO:0099543 | BP | trans-synaptic signaling by soluble gas | 3/18359 | 0.0222214 |  |
| 1 | GO:0110112 | BP | regulation of lipid transporter activity | 3/18359 | 0.0222214 |  |
| 1 | GO:0061113 | BP | pancreas morphogenesis | 3/18359 | 0.0222214 |  |
| 1 | GO:0060086 | BP | circadian temperature homeostasis | 3/18359 | 0.0222214 |  |
| 1 | GO:0070425 | BP | negative regulation of nucleotide-binding oligomerization domain containing signaling pathway | 3/18359 | 0.0222214 |  |
| 1 | GO:0150019 | BP | basal dendrite morphogenesis | 3/18359 | 0.0222214 |  |
| 1 | GO:0021825 | BP | substrate-dependent cerebral cortex tangential migration | 3/18359 | 0.0222214 |  |
| 1 | GO:0021824 | BP | cerebral cortex tangential migration using cell-axon interactions | 3/18359 | 0.0222214 |  |
| 1 | GO:0021828 | BP | gonadotrophin-releasing hormone neuronal migration to the hypothalamus | 3/18359 | 0.0222214 |  |
| 1 | GO:0060221 | BP | retinal rod cell differentiation | 3/18359 | 0.0222214 |  |
| 1 | GO:0016344 | BP | meiotic chromosome movement towards spindle pole | 3/18359 | 0.0222214 |  |
| 1 | GO:0150020 | BP | basal dendrite arborization | 3/18359 | 0.0222214 |  |
| 1 | GO:1990164 | BP | histone H2A phosphorylation | 3/18359 | 0.0222214 |  |
| 1 | GO:0099163 | BP | synaptic signaling by nitric oxide | 3/18359 | 0.0222214 |  |
| 2 | GO:0050686 | BP | negative regulation of mRNA processing | 31/18359 | 0.022317386 |  |
| 2 | GO:0043537 | BP | negative regulation of blood vessel endothelial cell migration | 31/18359 | 0.022317386 |  |
| 4 | GO:1902904 | BP | negative regulation of supramolecular fiber organization | 143/18359 | 0.022359287 |  |
| 3 | GO:0055002 | BP | striated muscle cell development | 81/18359 | 0.022730543 |  |
| 3 | GO:0060402 | BP | calcium ion transport into cytosol | 81/18359 | 0.022730543 |  |
| 4 | GO:0035023 | BP | regulation of Rho protein signal transduction | 145/18359 | 0.023385646 |  |
| 2 | GO:0018279 | BP | protein N-linked glycosylation via asparagine | 32/18359 | 0.023690481 |  |
| 2 | GO:0030049 | BP | muscle filament sliding | 32/18359 | 0.023690481 |  |
| 2 | GO:0010762 | BP | regulation of fibroblast migration | 32/18359 | 0.023690481 |  |
| 2 | GO:0033275 | BP | actin-myosin filament sliding | 32/18359 | 0.023690481 |  |
| 2 | GO:0051647 | BP | nucleus localization | 32/18359 | 0.023690481 |  |
| 7 | GO:0015672 | BP | monovalent inorganic cation transport | 383/18359 | 0.024933089 |  |
| 3 | GO:1903312 | BP | negative regulation of mRNA metabolic process | 84/18359 | 0.024981641 |  |
| 2 | GO:0042407 | BP | cristae formation | 33/18359 | 0.025097428 |  |
| 2 | GO:0046320 | BP | regulation of fatty acid oxidation | 33/18359 | 0.025097428 |  |
| 2 | GO:0018196 | BP | peptidyl-asparagine modification | 33/18359 | 0.025097428 |  |
| 3 | GO:0032204 | BP | regulation of telomere maintenance | 85/18359 | 0.025757782 |  |
| 13 | GO:1901698 | BP | response to nitrogen compound | 909/18359 | 0.026040125 |  |
| 5 | GO:0009790 | BP | embryo development | 224/18359 | 0.026447294 |  |
| 5 | GO:0009792 | BP | embryo development ending in birth or egg hatching | 224/18359 | 0.026447294 |  |
| 2 | GO:0055013 | BP | cardiac muscle cell development | 34/18359 | 0.026537654 |  |
| 2 | GO:0010464 | BP | regulation of mesenchymal cell proliferation | 34/18359 | 0.026537654 |  |
| 6 | GO:0051961 | BP | negative regulation of nervous system development | 304/18359 | 0.026624193 |  |
| 15 | GO:0009628 | BP | response to abiotic stimulus | 1100/18359 | 0.027150977 |  |
| 4 | GO:0051494 | BP | negative regulation of cytoskeleton organization | 153/18359 | 0.02777509 |  |
| 2 | GO:0051973 | BP | positive regulation of telomerase activity | 35/18359 | 0.028010592 |  |
| 13 | GO:0051960 | BP | regulation of nervous system development | 928/18359 | 0.028119573 |  |
| 5 | GO:0008016 | BP | regulation of heart contraction | 229/18359 | 0.028717253 |  |
| 4 | GO:0000904 | BP | cell morphogenesis involved in differentiation | 155/18359 | 0.028944131 |  |
| 2 | GO:0045923 | BP | positive regulation of fatty acid metabolic process | 36/18359 | 0.029515681 |  |
| 1 | GO:0010750 | BP | positive regulation of nitric oxide mediated signal transduction | 4/18359 | 0.029519051 |  |
| 1 | GO:0035616 | BP | histone H2B conserved C-terminal lysine deubiquitination | 4/18359 | 0.029519051 |  |
| 1 | GO:0021855 | BP | hypothalamus cell migration | 4/18359 | 0.029519051 |  |
| 1 | GO:0072553 | BP | terminal button organization | 4/18359 | 0.029519051 |  |
| 1 | GO:0097491 | BP | sympathetic neuron projection guidance | 4/18359 | 0.029519051 |  |
| 1 | GO:0097490 | BP | sympathetic neuron projection extension | 4/18359 | 0.029519051 |  |
| 1 | GO:0006011 | BP | UDP-glucose metabolic process | 4/18359 | 0.029519051 |  |
| 1 | GO:0046368 | BP | GDP-L-fucose metabolic process | 4/18359 | 0.029519051 |  |
| 1 | GO:0014809 | BP | regulation of skeletal muscle contraction by regulation of release of sequestered calcium ion | 4/18359 | 0.029519051 |  |
| 1 | GO:0072674 | BP | multinuclear osteoclast differentiation | 4/18359 | 0.029519051 |  |
| 1 | GO:0034155 | BP | regulation of toll-like receptor 7 signaling pathway | 4/18359 | 0.029519051 |  |
| 1 | GO:1902071 | BP | regulation of hypoxia-inducible factor-1alpha signaling pathway | 4/18359 | 0.029519051 |  |
| 1 | GO:0032185 | BP | septin cytoskeleton organization | 4/18359 | 0.029519051 |  |
| 1 | GO:0036486 | BP | ventral trunk neural crest cell migration | 4/18359 | 0.029519051 |  |
| 1 | GO:0036484 | BP | trunk neural crest cell migration | 4/18359 | 0.029519051 |  |
| 1 | GO:0034165 | BP | positive regulation of toll-like receptor 9 signaling pathway | 4/18359 | 0.029519051 |  |
| 1 | GO:0033059 | BP | cellular pigmentation | 4/18359 | 0.029519051 |  |
| 1 | GO:0071847 | BP | TNFSF11-mediated signaling pathway | 4/18359 | 0.029519051 |  |
| 1 | GO:2000566 | BP | positive regulation of CD8-positive, alpha-beta T cell proliferation | 4/18359 | 0.029519051 |  |
| 1 | GO:0071663 | BP | positive regulation of granzyme B production | 4/18359 | 0.029519051 |  |
| 1 | GO:0071661 | BP | regulation of granzyme B production | 4/18359 | 0.029519051 |  |
| 1 | GO:0008065 | BP | establishment of blood-nerve barrier | 4/18359 | 0.029519051 |  |
| 1 | GO:0060672 | BP | epithelial cell morphogenesis involved in placental branching | 4/18359 | 0.029519051 |  |
| 1 | GO:0090038 | BP | negative regulation of protein kinase C signaling | 4/18359 | 0.029519051 |  |
| 1 | GO:0051295 | BP | establishment of meiotic spindle localization | 4/18359 | 0.029519051 |  |
| 1 | GO:0032800 | BP | receptor biosynthetic process | 4/18359 | 0.029519051 |  |
| 1 | GO:0043932 | BP | ossification involved in bone remodeling | 4/18359 | 0.029519051 |  |
| 1 | GO:0045337 | BP | farnesyl diphosphate biosynthetic process | 4/18359 | 0.029519051 |  |
| 1 | GO:0000821 | BP | regulation of arginine metabolic process | 4/18359 | 0.029519051 |  |
| 1 | GO:0048880 | BP | sensory system development | 4/18359 | 0.029519051 |  |
| 3 | GO:0055001 | BP | muscle cell development | 90/18359 | 0.02983133 |  |
| 4 | GO:0008360 | BP | regulation of cell shape | 158/18359 | 0.030751919 |  |
| 2 | GO:0055006 | BP | cardiac cell development | 37/18359 | 0.031052366 |  |
| 5 | GO:0032271 | BP | regulation of protein polymerization | 234/18359 | 0.031107344 |  |
| 4 | GO:0051092 | BP | positive regulation of NF-kappaB transcription factor activity | 159/18359 | 0.031369021 |  |
| 3 | GO:0043535 | BP | regulation of blood vessel endothelial cell migration | 92/18359 | 0.031550441 |  |
| 3 | GO:0006639 | BP | acylglycerol metabolic process | 92/18359 | 0.031550441 |  |
| 3 | GO:0006638 | BP | neutral lipid metabolic process | 92/18359 | 0.031550441 |  |
| 2 | GO:0009225 | BP | nucleotide-sugar metabolic process | 38/18359 | 0.032620099 |  |
| 4 | GO:0030522 | BP | intracellular receptor signaling pathway | 161/18359 | 0.032625049 |  |
| 3 | GO:0002027 | BP | regulation of heart rate | 94/18359 | 0.033320549 |  |
| 28 | GO:0051128 | BP | regulation of cellular component organization | 2533/18359 | 0.033338075 |  |
| 2 | GO:0030501 | BP | positive regulation of bone mineralization | 39/18359 | 0.03421834 |  |
| 2 | GO:0048477 | BP | oogenesis | 39/18359 | 0.03421834 |  |
| 2 | GO:0048713 | BP | regulation of oligodendrocyte differentiation | 39/18359 | 0.03421834 |  |
| 2 | GO:0006754 | BP | ATP biosynthetic process | 39/18359 | 0.03421834 |  |
| 3 | GO:0006892 | BP | post-Golgi vesicle-mediated transport | 95/18359 | 0.034224662 |  |
| 6 | GO:0010721 | BP | negative regulation of cell development | 324/18359 | 0.034766912 |  |
| 4 | GO:0009260 | BP | ribonucleotide biosynthetic process | 165/18359 | 0.035224638 |  |
| 15 | GO:0040011 | BP | locomotion | 1177/18359 | 0.035597056 |  |
| 2 | GO:2001222 | BP | regulation of neuron migration | 40/18359 | 0.035846552 |  |
| 3 | GO:0014706 | BP | striated muscle tissue development | 97/18359 | 0.036070858 |  |
| 3 | GO:0060401 | BP | cytosolic calcium ion transport | 97/18359 | 0.036070858 |  |
| 1 | GO:0060666 | BP | dichotomous subdivision of terminal units involved in salivary gland branching | 5/18359 | 0.036762629 |  |
| 1 | GO:0060856 | BP | establishment of blood-brain barrier | 5/18359 | 0.036762629 |  |
| 1 | GO:0036124 | BP | histone H3-K9 trimethylation | 5/18359 | 0.036762629 |  |
| 1 | GO:0098735 | BP | positive regulation of the force of heart contraction | 5/18359 | 0.036762629 |  |
| 1 | GO:1904753 | BP | negative regulation of vascular associated smooth muscle cell migration | 5/18359 | 0.036762629 |  |
| 1 | GO:0021637 | BP | trigeminal nerve structural organization | 5/18359 | 0.036762629 |  |
| 1 | GO:0061551 | BP | trigeminal ganglion development | 5/18359 | 0.036762629 |  |
| 1 | GO:0061550 | BP | cranial ganglion development | 5/18359 | 0.036762629 |  |
| 1 | GO:0021785 | BP | branchiomotor neuron axon guidance | 5/18359 | 0.036762629 |  |
| 1 | GO:0014722 | BP | regulation of skeletal muscle contraction by calcium ion signaling | 5/18359 | 0.036762629 |  |
| 1 | GO:0070291 | BP | N-acylethanolamine metabolic process | 5/18359 | 0.036762629 |  |
| 1 | GO:0060040 | BP | retinal bipolar neuron differentiation | 5/18359 | 0.036762629 |  |
| 1 | GO:1901537 | BP | positive regulation of DNA demethylation | 5/18359 | 0.036762629 |  |
| 1 | GO:0006798 | BP | polyphosphate catabolic process | 5/18359 | 0.036762629 |  |
| 1 | GO:0035547 | BP | regulation of interferon-beta secretion | 5/18359 | 0.036762629 |  |
| 1 | GO:0035549 | BP | positive regulation of interferon-beta secretion | 5/18359 | 0.036762629 |  |
| 1 | GO:1901628 | BP | positive regulation of postsynaptic membrane organization | 5/18359 | 0.036762629 |  |
| 1 | GO:0022027 | BP | interkinetic nuclear migration | 5/18359 | 0.036762629 |  |
| 1 | GO:0033239 | BP | negative regulation of cellular amine metabolic process | 5/18359 | 0.036762629 |  |
| 1 | GO:0042270 | BP | protection from natural killer cell mediated cytotoxicity | 5/18359 | 0.036762629 |  |
| 1 | GO:0043987 | BP | histone H3-S10 phosphorylation | 5/18359 | 0.036762629 |  |
| 1 | GO:1904393 | BP | regulation of skeletal muscle acetylcholine-gated channel clustering | 5/18359 | 0.036762629 |  |
| 1 | GO:1904398 | BP | positive regulation of neuromuscular junction development | 5/18359 | 0.036762629 |  |
| 1 | GO:1905563 | BP | negative regulation of vascular endothelial cell proliferation | 5/18359 | 0.036762629 |  |
| 1 | GO:0040038 | BP | polar body extrusion after meiotic divisions | 5/18359 | 0.036762629 |  |
| 1 | GO:0042494 | BP | detection of bacterial lipoprotein | 5/18359 | 0.036762629 |  |
| 1 | GO:0097368 | BP | establishment of Sertoli cell barrier | 5/18359 | 0.036762629 |  |
| 1 | GO:0007406 | BP | negative regulation of neuroblast proliferation | 5/18359 | 0.036762629 |  |
| 1 | GO:0045338 | BP | farnesyl diphosphate metabolic process | 5/18359 | 0.036762629 |  |
| 1 | GO:0010891 | BP | negative regulation of sequestering of triglyceride | 5/18359 | 0.036762629 |  |
| 1 | GO:0002232 | BP | leukocyte chemotaxis involved in inflammatory response | 5/18359 | 0.036762629 |  |
| 3 | GO:0043112 | BP | receptor metabolic process | 98/18359 | 0.037012877 |  |
| 2 | GO:2000279 | BP | negative regulation of DNA biosynthetic process | 41/18359 | 0.037504207 |  |
| 2 | GO:0042220 | BP | response to cocaine | 41/18359 | 0.037504207 |  |
| 4 | GO:0046390 | BP | ribose phosphate biosynthetic process | 170/18359 | 0.038638789 |  |
| 3 | GO:0007179 | BP | transforming growth factor beta receptor signaling pathway | 100/18359 | 0.038934587 |  |
| 4 | GO:0007156 | BP | homophilic cell adhesion via plasma membrane adhesion molecules | 172/18359 | 0.040055792 |  |
| 5 | GO:0000902 | BP | cell morphogenesis | 252/18359 | 0.04072859 |  |
| 4 | GO:0009410 | BP | response to xenobiotic stimulus | 173/18359 | 0.040775306 |  |
| 2 | GO:0070252 | BP | actin-mediated cell contraction | 43/18359 | 0.040905757 |  |
| 3 | GO:0045807 | BP | positive regulation of endocytosis | 102/18359 | 0.040906274 |  |
| 5 | GO:0046578 | BP | regulation of Ras protein signal transduction | 253/18359 | 0.041310484 |  |
| 6 | GO:1903829 | BP | positive regulation of cellular protein localization | 338/18359 | 0.041333121 |  |
| 2 | GO:0060048 | BP | cardiac muscle contraction | 44/18359 | 0.042648625 |  |
| 2 | GO:0016358 | BP | dendrite development | 44/18359 | 0.042648625 |  |
| 2 | GO:0001895 | BP | retina homeostasis | 44/18359 | 0.042648625 |  |
| 4 | GO:0030833 | BP | regulation of actin filament polymerization | 176/18359 | 0.0429779 |  |
| 1 | GO:0034773 | BP | histone H4-K20 trimethylation | 6/18359 | 0.043952535 |  |
| 1 | GO:0019919 | BP | peptidyl-arginine methylation, to asymmetrical-dimethyl arginine | 6/18359 | 0.043952535 |  |
| 1 | GO:0030046 | BP | parallel actin filament bundle assembly | 6/18359 | 0.043952535 |  |
| 1 | GO:0010871 | BP | negative regulation of receptor biosynthetic process | 6/18359 | 0.043952535 |  |
| 1 | GO:0090230 | BP | regulation of centromere complex assembly | 6/18359 | 0.043952535 |  |
| 1 | GO:0070432 | BP | regulation of nucleotide-binding oligomerization domain containing 2 signaling pathway | 6/18359 | 0.043952535 |  |
| 1 | GO:0043152 | BP | induction of bacterial agglutination | 6/18359 | 0.043952535 |  |
| 1 | GO:0052572 | BP | response to host immune response | 6/18359 | 0.043952535 |  |
| 1 | GO:1905144 | BP | response to acetylcholine | 6/18359 | 0.043952535 |  |
| 1 | GO:0010742 | BP | macrophage derived foam cell differentiation | 6/18359 | 0.043952535 |  |
| 1 | GO:1901166 | BP | neural crest cell migration involved in autonomic nervous system development | 6/18359 | 0.043952535 |  |
| 1 | GO:0060350 | BP | endochondral bone morphogenesis | 6/18359 | 0.043952535 |  |
| 1 | GO:0052200 | BP | response to host defenses | 6/18359 | 0.043952535 |  |
| 1 | GO:0006797 | BP | polyphosphate metabolic process | 6/18359 | 0.043952535 |  |
| 1 | GO:0060385 | BP | axonogenesis involved in innervation | 6/18359 | 0.043952535 |  |
| 1 | GO:0075136 | BP | response to host | 6/18359 | 0.043952535 |  |
| 1 | GO:1903243 | BP | negative regulation of cardiac muscle hypertrophy in response to stress | 6/18359 | 0.043952535 |  |
| 1 | GO:1900454 | BP | positive regulation of long-term synaptic depression | 6/18359 | 0.043952535 |  |
| 1 | GO:0038203 | BP | TORC2 signaling | 6/18359 | 0.043952535 |  |
| 1 | GO:0010616 | BP | negative regulation of cardiac muscle adaptation | 6/18359 | 0.043952535 |  |
| 1 | GO:2000481 | BP | positive regulation of cAMP-dependent protein kinase activity | 6/18359 | 0.043952535 |  |
| 1 | GO:2000483 | BP | negative regulation of interleukin-8 secretion | 6/18359 | 0.043952535 |  |
| 1 | GO:0052173 | BP | response to defenses of other organism involved in symbiotic interaction | 6/18359 | 0.043952535 |  |
| 1 | GO:0052564 | BP | response to immune response of other organism involved in symbiotic interaction | 6/18359 | 0.043952535 |  |
| 1 | GO:1903753 | BP | negative regulation of p38MAPK cascade | 6/18359 | 0.043952535 |  |
| 1 | GO:0051599 | BP | response to hydrostatic pressure | 6/18359 | 0.043952535 |  |
| 1 | GO:0090077 | BP | foam cell differentiation | 6/18359 | 0.043952535 |  |
| 1 | GO:0021902 | BP | commitment of neuronal cell to specific neuron type in forebrain | 6/18359 | 0.043952535 |  |
| 1 | GO:0007185 | BP | transmembrane receptor protein tyrosine phosphatase signaling pathway | 6/18359 | 0.043952535 |  |
| 1 | GO:0030252 | BP | growth hormone secretion | 6/18359 | 0.043952535 |  |
| 1 | GO:0006207 | BP | 'de novo' pyrimidine nucleobase biosynthetic process | 6/18359 | 0.043952535 |  |
| 2 | GO:1900271 | BP | regulation of long-term synaptic potentiation | 45/18359 | 0.044418879 |  |
| 2 | GO:0070509 | BP | calcium ion import | 45/18359 | 0.044418879 |  |
| 2 | GO:0048662 | BP | negative regulation of smooth muscle cell proliferation | 45/18359 | 0.044418879 |  |
| 3 | GO:0060359 | BP | response to ammonium ion | 106/18359 | 0.044998269 |  |
| 3 | GO:1903076 | BP | regulation of protein localization to plasma membrane | 106/18359 | 0.044998269 |  |
| 3 | GO:0048024 | BP | regulation of mRNA splicing, via spliceosome | 107/18359 | 0.046051972 |  |
| 2 | GO:0070169 | BP | positive regulation of biomineral tissue development | 46/18359 | 0.046216021 |  |
| 2 | GO:0035722 | BP | interleukin-12-mediated signaling pathway | 46/18359 | 0.046216021 |  |
| 21 | GO:1902531 | BP | regulation of intracellular signal transduction | 1858/18359 | 0.04678191 |  |
| 3 | GO:0022904 | BP | respiratory electron transport chain | 108/18359 | 0.047117862 |  |
| 5 | GO:0098742 | BP | cell-cell adhesion via plasma-membrane adhesion molecules | 263/18359 | 0.047407356 |  |
| 2 | GO:0045687 | BP | positive regulation of glial cell differentiation | 47/18359 | 0.048039556 |  |
| 2 | GO:1904705 | BP | regulation of vascular smooth muscle cell proliferation | 47/18359 | 0.048039556 |  |
| 9 | GO:0098655 | BP | cation transmembrane transport | 607/18359 | 0.048127411 |  |
| 28 | GO:0010605 | BP | negative regulation of macromolecule metabolic process | 2627/18359 | 0.049017233 |  |
| 2 | GO:0045104 | BP | intermediate filament cytoskeleton organization | 48/18359 | 0.049888997 |  |
| 2 | GO:0002673 | BP | regulation of acute inflammatory response | 48/18359 | 0.049888997 |  |
| 7 | GO:0051090 | BP | regulation of DNA-binding transcription factor activity | 446/18359 | 0.049967638 |  |
| 4 | GO:0000302 | BP | response to reactive oxygen species | 185/18359 | 0.049981772 |  |
| 9 | GO:0030424 | CC | axon | 396/18359 | 0.002927525 |  |
| 7 | GO:0015629 | CC | actin cytoskeleton | 260/18359 | 0.0034054 |  |
| 7 | GO:0098794 | CC | postsynapse | 271/18359 | 0.004269871 |  |
| 5 | GO:0098800 | CC | inner mitochondrial membrane protein complex | 146/18359 | 0.004792003 |  |
| 2 | GO:0099059 | CC | integral component of presynaptic active zone membrane | 15/18359 | 0.005446564 |  |
| 2 | GO:0043034 | CC | costamere | 17/18359 | 0.006986131 |  |
| 1 | GO:0070319 | CC | Golgi to plasma membrane transport vesicle | 1/18359 | 0.00746228 |  |
| 1 | GO:0005586 | CC | collagen type III trimer | 1/18359 | 0.00746228 |  |
| 1 | GO:0034999 | CC | oligosaccharyltransferase II complex | 1/18359 | 0.00746228 |  |
| 4 | GO:0042383 | CC | sarcolemma | 104/18359 | 0.007676564 |  |
| 9 | GO:0005911 | CC | cell-cell junction | 464/18359 | 0.008097645 |  |
| 2 | GO:0098945 | CC | intrinsic component of presynaptic active zone membrane | 19/18359 | 0.008698882 |  |
| 2 | GO:0045259 | CC | proton-transporting ATP synthase complex | 20/18359 | 0.009618505 |  |
| 2 | GO:0005753 | CC | mitochondrial proton-transporting ATP synthase complex | 20/18359 | 0.009618505 |  |
| 22 | GO:0120025 | CC | plasma membrane bounded cell projection | 1738/18359 | 0.012282692 |  |
| 2 | GO:0071556 | CC | integral component of lumenal side of endoplasmic reticulum membrane | 24/18359 | 0.013703208 |  |
| 1 | GO:0008074 | CC | guanylate cyclase complex, soluble | 2/18359 | 0.014869278 |  |
| 1 | GO:0034998 | CC | oligosaccharyltransferase I complex | 2/18359 | 0.014869278 |  |
| 1 | GO:0071575 | CC | integral component of external side of plasma membrane | 2/18359 | 0.014869278 |  |
| 3 | GO:0070469 | CC | respirasome | 73/18359 | 0.017294748 |  |
| 15 | GO:0043005 | CC | neuron projection | 1085/18359 | 0.017697804 |  |
| 3 | GO:0031594 | CC | neuromuscular junction | 74/18359 | 0.017929182 |  |
| 2 | GO:0002102 | CC | podosome | 28/18359 | 0.018407056 |  |
| 1 | GO:0099617 | CC | matrix side of mitochondrial inner membrane | 3/18359 | 0.0222214 |  |
| 1 | GO:1990761 | CC | growth cone lamellipodium | 3/18359 | 0.0222214 |  |
| 3 | GO:0005884 | CC | actin filament | 83/18359 | 0.024218383 |  |
| 23 | GO:0042995 | CC | cell projection | 1952/18359 | 0.025200713 |  |
| 35 | GO:0043232 | CC | intracellular non-membrane-bounded organelle | 3305/18359 | 0.025314618 |  |
| 35 | GO:0043228 | CC | non-membrane-bounded organelle | 3312/18359 | 0.025589122 |  |
| 50 | GO:0005829 | CC | cytosol | 5075/18359 | 0.02691319 |  |
| 1 | GO:0000938 | CC | GARP complex | 4/18359 | 0.029519051 |  |
| 1 | GO:0016013 | CC | syntrophin complex | 4/18359 | 0.029519051 |  |
| 1 | GO:0042721 | CC | TIM22 mitochondrial import inner membrane insertion complex | 4/18359 | 0.029519051 |  |
| 1 | GO:0000275 | CC | mitochondrial proton-transporting ATP synthase complex, catalytic core F(1) | 4/18359 | 0.029519051 |  |
| 17 | GO:0030054 | CC | cell junction | 1359/18359 | 0.032104965 |  |
| 104 | GO:0043226 | CC | organelle | 12395/18359 | 0.035256344 |  |
| 7 | GO:0030055 | CC | cell-substrate junction | 416/18359 | 0.036614577 |  |
| 1 | GO:0070695 | CC | FHF complex | 5/18359 | 0.036762629 |  |
| 1 | GO:0098871 | CC | postsynaptic actin cytoskeleton | 5/18359 | 0.036762629 |  |
| 1 | GO:0045261 | CC | proton-transporting ATP synthase complex, catalytic core F(1) | 5/18359 | 0.036762629 |  |
| 2 | GO:0016469 | CC | proton-transporting two-sector ATPase complex | 41/18359 | 0.037504207 |  |
| 2 | GO:0030315 | CC | T-tubule | 44/18359 | 0.042648625 |  |
| 3 | GO:0016363 | CC | nuclear matrix | 104/18359 | 0.042927617 |  |
| 1 | GO:0098576 | CC | lumenal side of membrane | 6/18359 | 0.043952535 |  |
| 1 | GO:0071546 | CC | pi-body | 6/18359 | 0.043952535 |  |
| 2 | GO:0014704 | CC | intercalated disc | 45/18359 | 0.044418879 |  |
| 2 | GO:0030016 | CC | myofibril | 45/18359 | 0.044418879 |  |
| 2 | GO:0005637 | CC | nuclear inner membrane | 48/18359 | 0.049888997 |  |
| 3 | GO:0042805 | MF | actinin binding | 44/18359 | 0.004305533 |  |
| 5 | GO:0005178 | MF | integrin binding | 143/18359 | 0.004390065 |  |
| 2 | GO:0050998 | MF | nitric-oxide synthase binding | 14/18359 | 0.00474345 |  |
| 3 | GO:0098531 | MF | ligand-activated transcription factor activity | 49/18359 | 0.005829645 |  |
| 3 | GO:0004879 | MF | nuclear receptor activity | 49/18359 | 0.005829645 |  |
| 6 | GO:0005085 | MF | guanyl-nucleotide exchange factor activity | 219/18359 | 0.006062957 |  |
| 1 | GO:0036487 | MF | nitric-oxide synthase inhibitor activity | 1/18359 | 0.00746228 |  |
| 1 | GO:0004588 | MF | orotate phosphoribosyltransferase activity | 1/18359 | 0.00746228 |  |
| 1 | GO:0033925 | MF | mannosyl-glycoprotein endo-beta-N-acetylglucosaminidase activity | 1/18359 | 0.00746228 |  |
| 1 | GO:0035375 | MF | zymogen binding | 1/18359 | 0.00746228 |  |
| 1 | GO:0047395 | MF | glycerophosphoinositol glycerophosphodiesterase activity | 1/18359 | 0.00746228 |  |
| 1 | GO:0051748 | MF | UTP-monosaccharide-1-phosphate uridylyltransferase activity | 1/18359 | 0.00746228 |  |
| 1 | GO:0046921 | MF | alpha-(1->6)-fucosyltransferase activity | 1/18359 | 0.00746228 |  |
| 1 | GO:0035642 | MF | histone methyltransferase activity (H3-R17 specific) | 1/18359 | 0.00746228 |  |
| 1 | GO:0004590 | MF | orotidine-5'-phosphate decarboxylase activity | 1/18359 | 0.00746228 |  |
| 1 | GO:0003983 | MF | UTP:glucose-1-phosphate uridylyltransferase activity | 1/18359 | 0.00746228 |  |
| 1 | GO:0008424 | MF | glycoprotein 6-alpha-L-fucosyltransferase activity | 1/18359 | 0.00746228 |  |
| 3 | GO:0003707 | MF | steroid hormone receptor activity | 56/18359 | 0.008445724 |  |
| 2 | GO:0046933 | MF | proton-transporting ATP synthase activity, rotational mechanism | 20/18359 | 0.009618505 |  |
| 3 | GO:0048306 | MF | calcium-dependent protein binding | 63/18359 | 0.011651479 |  |
| 2 | GO:0050321 | MF | tau-protein kinase activity | 24/18359 | 0.013703208 |  |
| 5 | GO:0005516 | MF | calmodulin binding | 191/18359 | 0.014335098 |  |
| 12 | GO:0008289 | MF | lipid binding | 761/18359 | 0.014593303 |  |
| 1 | GO:0008889 | MF | glycerophosphodiester phosphodiesterase activity | 2/18359 | 0.014869278 |  |
| 1 | GO:0099580 | MF | ion antiporter activity involved in regulation of postsynaptic membrane potential | 2/18359 | 0.014869278 |  |
| 1 | GO:1905060 | MF | calcium:cation antiporter activity involved in regulation of postsynaptic cytosolic calcium ion concentration | 2/18359 | 0.014869278 |  |
| 1 | GO:0004692 | MF | cGMP-dependent protein kinase activity | 2/18359 | 0.014869278 |  |
| 1 | GO:0004421 | MF | hydroxymethylglutaryl-CoA synthase activity | 2/18359 | 0.014869278 |  |
| 3 | GO:0019829 | MF | ATPase-coupled cation transmembrane transporter activity | 72/18359 | 0.016673141 |  |
| 4 | GO:0005088 | MF | Ras guanyl-nucleotide exchange factor activity | 131/18359 | 0.016784374 |  |
| 3 | GO:0033293 | MF | monocarboxylic acid binding | 74/18359 | 0.017929182 |  |
| 5 | GO:0008022 | MF | protein C-terminus binding | 203/18359 | 0.01818271 |  |
| 3 | GO:0005089 | MF | Rho guanyl-nucleotide exchange factor activity | 76/18359 | 0.0192366 |  |
| 3 | GO:0042625 | MF | ATPase-coupled ion transmembrane transporter activity | 76/18359 | 0.0192366 |  |
| 8 | GO:0005543 | MF | phospholipid binding | 450/18359 | 0.019732948 |  |
| 3 | GO:0038024 | MF | cargo receptor activity | 79/18359 | 0.021294289 |  |
| 5 | GO:0051015 | MF | actin filament binding | 213/18359 | 0.021867394 |  |
| 1 | GO:0070137 | MF | ubiquitin-like protein-specific endopeptidase activity | 3/18359 | 0.0222214 |  |
| 1 | GO:0070139 | MF | SUMO-specific endopeptidase activity | 3/18359 | 0.0222214 |  |
| 1 | GO:0035373 | MF | chondroitin sulfate proteoglycan binding | 3/18359 | 0.0222214 |  |
| 3 | GO:0005546 | MF | phosphatidylinositol-4,5-bisphosphate binding | 82/18359 | 0.023468016 |  |
| 4 | GO:0019903 | MF | protein phosphatase binding | 147/18359 | 0.024440258 |  |
| 2 | GO:0044769 | MF | ATPase activity, coupled to transmembrane movement of ions, rotational mechanism | 36/18359 | 0.029515681 |  |
| 2 | GO:0001103 | MF | RNA polymerase II repressing transcription factor binding | 36/18359 | 0.029515681 |  |
| 1 | GO:0030943 | MF | mitochondrion targeting sequence binding | 4/18359 | 0.029519051 |  |
| 1 | GO:0050692 | MF | DBD domain binding | 4/18359 | 0.029519051 |  |
| 1 | GO:0016286 | MF | small conductance calcium-activated potassium channel activity | 4/18359 | 0.029519051 |  |
| 2 | GO:0031492 | MF | nucleosomal DNA binding | 37/18359 | 0.031052366 |  |
| 2 | GO:0008307 | MF | structural constituent of muscle | 37/18359 | 0.031052366 |  |
| 2 | GO:0048156 | MF | tau protein binding | 39/18359 | 0.03421834 |  |
| 2 | GO:0030020 | MF | extracellular matrix structural constituent conferring tensile strength | 39/18359 | 0.03421834 |  |
| 5 | GO:0005539 | MF | glycosaminoglycan binding | 241/18359 | 0.034658226 |  |
| 1 | GO:0070915 | MF | lysophosphatidic acid receptor activity | 5/18359 | 0.036762629 |  |
| 1 | GO:0070891 | MF | lipoteichoic acid binding | 5/18359 | 0.036762629 |  |
| 1 | GO:0004309 | MF | exopolyphosphatase activity | 5/18359 | 0.036762629 |  |
| 14 | GO:0044212 | MF | transcription regulatory region DNA binding | 1048/18359 | 0.03837533 |  |
| 14 | GO:0001067 | MF | regulatory region nucleic acid binding | 1048/18359 | 0.03837533 |  |
| 2 | GO:0005504 | MF | fatty acid binding | 42/18359 | 0.039190781 |  |
| 3 | GO:0031490 | MF | chromatin DNA binding | 104/18359 | 0.042927617 |  |
| 9 | GO:0003682 | MF | chromatin binding | 585/18359 | 0.043025061 |  |
| 1 | GO:0008510 | MF | sodium:bicarbonate symporter activity | 6/18359 | 0.043952535 |  |
| 1 | GO:1990446 | MF | U1 snRNP binding | 6/18359 | 0.043952535 |  |
| 1 | GO:0046977 | MF | TAP binding | 6/18359 | 0.043952535 |  |
| 1 | GO:1990050 | MF | phosphatidic acid transfer activity | 6/18359 | 0.043952535 |  |
| 1 | GO:0016929 | MF | SUMO-specific protease activity | 6/18359 | 0.043952535 |  |
| 1 | GO:0046912 | MF | transferase activity, transferring acyl groups, acyl groups converted into alkyl on transfer | 6/18359 | 0.043952535 |  |
| 2 | GO:0004715 | MF | non-membrane spanning protein tyrosine kinase activity | 46/18359 | 0.046216021 |  |
| 5 | GO:0008017 | MF | microtubule binding | 262/18359 | 0.046774858 |  |
| 2 | GO:0003954 | MF | NADH dehydrogenase activity | 47/18359 | 0.048039556 |  |
| 2 | GO:0050136 | MF | NADH dehydrogenase (quinone) activity | 47/18359 | 0.048039556 |  |
| 2 | GO:0008137 | MF | NADH dehydrogenase (ubiquinone) activity | 47/18359 | 0.048039556 |  |
| 134 | GO:0003674 | MF | molecular_function | 17231/18359 | 0.04904958 |  |
| 25 | GO:0019899 | MF | enzyme binding | 2272/18359 | 0.049205118 |  |
