# Supplementary material for: Characterization of Circular RNA Expression Profiles in Colon Specimens of Patients with Slow Transit Constipation
Source: Dis Markers. 2022 Jun 10;2022:3653363. doi: 10.1155/2022/3653363 (PMC9206760; doi:10.1155/2022/3653363)
Supplement: Supplementary 4 — Table S4: the KEGG pathway enrichment terms. [file 3653363.f4.docx]

| **Table S4. KEGG pathway enrichment terms.** | | | | | | | |
| --- | --- | --- | --- | --- | --- | --- | --- |
| **Number** | **Pathway id** | **Description** | **Ratio_in_pop** | **pvalue** | **First Category** | **Second Category** | |
| 5 | map05012 | Parkinson disease | 145/13789 | 0.005408266 | Human Diseases | Neurodegenerative disease | |
| 4 | map03320 | PPAR signaling pathway | 72/13789 | 0.002351923 | Organismal Systems | Endocrine system | |
| 4 | map05410 | Hypertrophic cardiomyopathy (HCM) | 88/13789 | 0.004850553 | Human Diseases | Cardiovascular disease | |
| 4 | map04970 | Salivary secretion | 86/13789 | 0.004469565 | Organismal Systems | Digestive system | |
| 5 | map00190 | Oxidative phosphorylation | 136/13789 | 0.004126908 | Metabolism | Energy metabolism | |
| 7 | map04714 | Thermogenesis | 233/13789 | 0.002240899 | Organismal Systems | Environmental adaptation | |
| 6 | map05414 | Dilated cardiomyopathy (DCM) | 167/13789 | 0.001902726 | Human Diseases | Cardiovascular disease | |
| 3 | map05412 | Arrhythmogenic right ventricular cardiomyopathy (ARVC) | 75/13789 | 0.020489687 | Human Diseases | Cardiovascular disease | |
| 6 | map05016 | Huntington disease | 274/13789 | 0.019862401 | Human Diseases | Neurodegenerative disease | |
| 4 | map04261 | Adrenergic signaling in cardiomyocytes | 147/13789 | 0.02750227 | Organismal Systems | Circulatory system | |
| 5 | map05416 | Viral myocarditis | 197/13789 | 0.018626728 | Human Diseases | Cardiovascular disease | |
| 3 | map04260 | Cardiac muscle contraction | 82/13789 | 0.025852603 | Organismal Systems | Circulatory system | |
| 3 | map04713 | Circadian entrainment | 99/13789 | 0.041698918 | Organismal Systems | Environmental adaptation | |
| 4 | map04022 | cGMP-PKG signaling pathway | 166/13789 | 0.040252194 | Environmental Information Processing | Signal transduction | |
| 3 | map04974 | Protein digestion and absorption | 94/13789 | 0.036627804 | Organismal Systems | Digestive system | |
| 2 | map00513 | Various types of N-glycan biosynthesis | 41/13789 | 0.040200291 | Metabolism | Glycan biosynthesis and metabolism | |
| 3 | map04380 | Osteoclast differentiation | 128/13789 | 0.077482388 | Organismal Systems | Development and regeneration | |
| 2 | map04730 | Long-term depression | 59/13789 | 0.076738792 | Organismal Systems | Nervous system | |
| 1 | map00072 | Synthesis and degradation of ketone bodies | 10/13789 | 0.074967363 | Metabolism | Lipid metabolism | |
| 2 | map00510 | N-Glycan biosynthesis | 52/13789 | 0.061558244 | Metabolism | Glycan biosynthesis and metabolism | |
| 2 | map04961 | Endocrine and other factor-regulated calcium reabsorption | 51/13789 | 0.059483715 | Organismal Systems | Excretory system | |
| 3 | map04611 | Platelet activation | 126/13789 | 0.074684616 | Organismal Systems | Immune system | |
| 4 | map05203 | Viral carcinogenesis | 198/13789 | 0.068176553 | Human Diseases | Cancer: overview | |
| 2 | map04978 | Mineral absorption | 57/13789 | 0.072288676 | Organismal Systems | Digestive system | |
| 4 | map04145 | Phagosome | 230/13789 | 0.103875753 | Cellular Processes | Transport and catabolism | |
| 2 | map04976 | Bile secretion | 68/13789 | 0.097760109 | Organismal Systems | Digestive system | |
| 5 | map04020 | Calcium signaling pathway | 333/13789 | 0.117097917 | Environmental Information Processing | Signal transduction | |
| 3 | map05140 | Leishmaniasis | 152/13789 | 0.114423831 | Human Diseases | Infectious disease: parasitic | |
| 4 | map04072 | Phospholipase D signaling pathway | 223/13789 | 0.095439584 | Environmental Information Processing | Signal transduction | |
| 1 | map00533 | Glycosaminoglycan biosynthesis - keratan sulfate | 14/13789 | 0.103370603 | Metabolism | Glycan biosynthesis and metabolism | |
| 5 | map05169 | Epstein-Barr virus infection | 330/13789 | 0.113867926 | Human Diseases | Infectious disease: viral | |
| 5 | map05010 | Alzheimer disease | 341/13789 | 0.125923297 | Human Diseases | Neurodegenerative disease | |
| 1 | map00511 | Other glycan degradation | 18/13789 | 0.130909619 | Metabolism | Glycan biosynthesis and metabolism | |
| 1 | map04964 | Proximal tubule bicarbonate reclamation | 25/13789 | 0.177101292 | Organismal Systems | Excretory system | |
| 1 | map00650 | Butanoate metabolism | 27/13789 | 0.189846291 | Metabolism | Carbohydrate metabolism | |
| 2 | map04070 | Phosphatidylinositol signaling system | 100/13789 | 0.181981882 | Environmental Information Processing | Signal transduction | |
| 2 | map04933 | AGE-RAGE signaling pathway in diabetic complications | 102/13789 | 0.187570408 | Human Diseases | Endocrine and metabolic disease | |
| 2 | map04972 | Pancreatic secretion | 98/13789 | 0.176419569 | Organismal Systems | Digestive system | |
| 1 | map04950 | Maturity onset diabetes of the young | 24/13789 | 0.170654509 | Human Diseases | Endocrine and metabolic disease | |
| 3 | map04141 | Protein processing in endoplasmic reticulum | 168/13789 | 0.14209687 | Genetic Information Processing | Folding, sorting and degradation | |
| 4 | map05130 | Pathogenic Escherichia coli infection | 282/13789 | 0.176047605 | Human Diseases | Infectious disease: bacterial | |
| 3 | map05320 | Autoimmune thyroid disease | 180/13789 | 0.16417384 | Human Diseases | Immune disease | |
| 4 | map04015 | Rap1 signaling pathway | 277/13789 | 0.168465339 | Environmental Information Processing | Signal transduction | |
| 2 | map04540 | Gap junction | 91/13789 | 0.157189815 | Cellular Processes | Cellular community - eukaryotes | |
| 3 | map05330 | Allograft rejection | 175/13789 | 0.154850288 | Human Diseases | Immune disease | |
| 3 | map05146 | Amoebiasis | 173/13789 | 0.151169672 | Human Diseases | Infectious disease: parasitic | |
| 1 | map00900 | Terpenoid backbone biosynthesis | 23/13789 | 0.164157696 | Metabolism | Metabolism of terpenoids and polyketides | |
| 1 | map00052 | Galactose metabolism | 31/13789 | 0.214752533 | Metabolism | Carbohydrate metabolism | |
| 4 | map05202 | Transcriptional misregulation in cancer | 323/13789 | 0.242088785 | Human Diseases | Cancer: overview | |
| 2 | map05143 | African trypanosomiasis | 111/13789 | 0.212987863 | Human Diseases | Infectious disease: parasitic | |
| 1 | map05216 | Thyroid cancer | 37/13789 | 0.250696431 | Human Diseases | Cancer: specific types | |
| 2 | map05340 | Primary immunodeficiency | 118/13789 | 0.232980614 | Human Diseases | Immune disease | |
| 2 | map04270 | Vascular smooth muscle contraction | 126/13789 | 0.255963826 | Organismal Systems | Circulatory system | |
| 1 | map00500 | Starch and sucrose metabolism | 35/13789 | 0.238900029 | Metabolism | Carbohydrate metabolism | |
| 1 | map04975 | Fat digestion and absorption | 35/13789 | 0.238900029 | Organismal Systems | Digestive system | |
| 3 | map05323 | Rheumatoid arthritis | 229/13789 | 0.262466243 | Human Diseases | Immune disease | |
| 1 | map00040 | Pentose and glucuronate interconversions | 33/13789 | 0.226919676 | Metabolism | Carbohydrate metabolism | |
| 1 | map04320 | Dorso-ventral axis formation | 33/13789 | 0.226919676 | Organismal Systems | Development and regeneration | |
| 2 | map04612 | Antigen processing and presentation | 135/13789 | 0.281872318 | Organismal Systems | Immune system | |
| 1 | map05219 | Bladder cancer | 41/13789 | 0.273748537 | Human Diseases | Cancer: specific types | |
| 3 | map04064 | NF-kappa B signaling pathway | 238/13789 | 0.281400928 | Environmental Information Processing | Signal transduction | |
| 3 | map05152 | Tuberculosis | 248/13789 | 0.302584788 | Human Diseases | Infectious disease: bacterial | |
| 2 | map04664 | Fc epsilon RI signaling pathway | 142/13789 | 0.301984248 | Organismal Systems | Immune system | |
| 1 | map00520 | Amino sugar and nucleotide sugar metabolism | 49/13789 | 0.317765679 | Metabolism | Carbohydrate metabolism | |
| 1 | map00280 | Valine, leucine and isoleucine degradation | 50/13789 | 0.323078567 | Metabolism | Amino acid metabolism | |
| 2 | map04723 | Retrograde endocannabinoid signaling | 146/13789 | 0.313438669 | Organismal Systems | Nervous system | |
| 2 | map04672 | Intestinal immune network for IgA production | 187/13789 | 0.427301463 | Organismal Systems | Immune system | |
| 1 | map04924 | Renin secretion | 70/13789 | 0.421131181 | Organismal Systems | Endocrine system | |
| 1 | map04520 | Adherens junction | 70/13789 | 0.421131181 | Cellular Processes | Cellular community - eukaryotes | |
| 4 | map04151 | PI3K-Akt signaling pathway | 423/13789 | 0.416954723 | Environmental Information Processing | Signal transduction | |
| 2 | map04650 | Natural killer cell mediated cytotoxicity | 178/13789 | 0.403035074 | Organismal Systems | Immune system | |
| 1 | map00562 | Inositol phosphate metabolism | 75/13789 | 0.443359131 | Metabolism | Carbohydrate metabolism | |
| 1 | map00983 | Drug metabolism - other enzymes | 74/13789 | 0.438982252 | Metabolism | Xenobiotics biodegradation and metabolism | |
| 2 | map04662 | B cell receptor signaling pathway | 155/13789 | 0.339055933 | Organismal Systems | Immune system | |
| 1 | map04137 | Mitophagy - animal | 66/13789 | 0.402717581 | Cellular Processes | Transport and catabolism | |
| 1 | map04929 | GnRH secretion | 65/13789 | 0.398024241 | Organismal Systems | Endocrine system | |
| 1 | map04918 | Thyroid hormone synthesis | 69/13789 | 0.416581195 | Organismal Systems | Endocrine system | |
| 1 | map04917 | Prolactin signaling pathway | 69/13789 | 0.416581195 | Organismal Systems | Endocrine system | |
| 2 | map05310 | Asthma | 168/13789 | 0.375529942 | Human Diseases | Immune disease | |
| 3 | map05166 | Human T-cell leukemia virus 1 infection | 280/13789 | 0.370600031 | Human Diseases | Infectious disease: viral | |
| 2 | map04530 | Tight junction | 170/13789 | 0.381073051 | Cellular Processes | Cellular community - eukaryotes | |
| 3 | map05165 | Human papillomavirus infection | 325/13789 | 0.463891697 | Human Diseases | Infectious disease: viral | |
| 2 | map04666 | Fc gamma R-mediated phagocytosis | 172/13789 | 0.386595801 | Organismal Systems | Immune system | |
| 2 | map05167 | Kaposi sarcoma-associated herpesvirus infection | 176/13789 | 0.397577469 | Human Diseases | Infectious disease: viral | |
| 1 | map05212 | Pancreatic cancer | 79/13789 | 0.460530926 | Human Diseases | Cancer: specific types | |
| 2 | map04640 | Hematopoietic cell lineage | 175/13789 | 0.394840239 | Organismal Systems | Immune system | |
| 1 | map00561 | Glycerolipid metabolism | 58/13789 | 0.364132034 | Metabolism | Lipid metabolism | |
| 1 | map00240 | Pyrimidine metabolism | 57/13789 | 0.359138428 | Metabolism | Nucleotide metabolism | |
| 1 | map04923 | Regulation of lipolysis in adipocytes | 56/13789 | 0.354105977 | Organismal Systems | Endocrine system | |
| 2 | map05206 | MicroRNAs in cancer | 166/13789 | 0.369967194 | Human Diseases | Cancer: overview | |
| 1 | map00564 | Glycerophospholipid metabolism | 97/13789 | 0.531533245 | Metabolism | Lipid metabolism | |
| 1 | map04361 | Axon regeneration | 92/13789 | 0.512795525 | Organismal Systems | Development and regeneration | |
| 2 | map05150 | Staphylococcus aureus infection | 223/13789 | 0.518834782 | Human Diseases | Infectious disease: bacterial | |
| 1 | map04911 | Insulin secretion | 83/13789 | 0.477177787 | Organismal Systems | Endocrine system | |
| 1 | map01521 | EGFR tyrosine kinase inhibitor resistance | 83/13789 | 0.477177787 | Human Diseases | Drug resistance: antineoplastic | |
| 1 | map04212 | Longevity regulating pathway - worm | 91/13789 | 0.508959834 | Organismal Systems | Aging |  |
| 1 | map04940 | Type I diabetes mellitus | 104/13789 | 0.556572325 | Human Diseases | Endocrine and metabolic disease | |
| 4 | map05200 | Pathways in cancer | 531/13789 | 0.594687886 | Human Diseases | Cancer: overview | |
| 1 | map05161 | Hepatitis B | 152/13789 | 0.695975792 | Human Diseases | Infectious disease: viral | |
| 2 | map05132 | Salmonella infection | 218/13789 | 0.506698393 | Human Diseases | Infectious disease: bacterial | |
| 1 | map05332 | Graft-versus-host disease | 96/13789 | 0.527843716 | Human Diseases | Immune disease | |
| 1 | map04925 | Aldosterone synthesis and secretion | 96/13789 | 0.527843716 | Organismal Systems | Endocrine system | |
| 1 | map05231 | Choline metabolism in cancer | 102/13789 | 0.549556796 | Human Diseases | Cancer: overview | |
| 1 | map05235 | PD-L1 expression and PD-1 checkpoint pathway in cancer | 155/13789 | 0.7030767 | Human Diseases | Cancer: overview | |
| 1 | map05145 | Toxoplasmosis | 113/13789 | 0.586826165 | Human Diseases | Infectious disease: parasitic | |
| 1 | map04668 | TNF signaling pathway | 113/13789 | 0.586826165 | Environmental Information Processing | Signal transduction | |
| 2 | map05131 | Shigellosis | 239/13789 | 0.556333635 | Human Diseases | Infectious disease: bacterial | |
| 1 | map04658 | Th1 and Th2 cell differentiation | 158/13789 | 0.710013271 | Organismal Systems | Immune system | |
| 2 | map04144 | Endocytosis | 251/13789 | 0.583089402 | Cellular Processes | Transport and catabolism | |
| 1 | map01522 | Endocrine resistance | 101/13789 | 0.54600791 | Human Diseases | Drug resistance: antineoplastic | |
| 4 | map05168 | Herpes simplex virus 1 infection | 517/13789 | 0.573361614 | Human Diseases | Infectious disease: viral | |
| 1 | map04659 | Th17 cell differentiation | 171/13789 | 0.738262937 | Organismal Systems | Immune system | |
| 1 | map04621 | NOD-like receptor signaling pathway | 166/13789 | 0.727735751 | Organismal Systems | Immune system | |
| 1 | map04926 | Relaxin signaling pathway | 126/13789 | 0.62695002 | Organismal Systems | Endocrine system | |
| 1 | map04211 | Longevity regulating pathway | 90/13789 | 0.505094229 | Organismal Systems | Aging |  |
| 1 | map04726 | Serotonergic synapse | 111/13789 | 0.580284934 | Organismal Systems | Nervous system | |
| 1 | map04218 | Cellular senescence | 162/13789 | 0.719012896 | Cellular Processes | Cell growth and death | |
| 1 | map04919 | Thyroid hormone signaling pathway | 123/13789 | 0.618047778 | Organismal Systems | Endocrine system | |
| 1 | map03013 | RNA transport | 170/13789 | 0.73619027 | Genetic Information Processing | Translation |  |
| 1 | map05164 | Influenza A | 165/13789 | 0.725580519 | Human Diseases | Infectious disease: viral | |
| 1 | map04630 | Jak-STAT signaling pathway | 151/13789 | 0.693571639 | Environmental Information Processing | Signal transduction | |
| 1 | map04217 | Necroptosis | 151/13789 | 0.693571639 | Cellular Processes | Cell growth and death | |
| 2 | map04014 | Ras signaling pathway | 299/13789 | 0.6782324 | Environmental Information Processing | Signal transduction | |
| 1 | map00230 | Purine metabolism | 129/13789 | 0.635646669 | Metabolism | Nucleotide metabolism | |
| 2 | map05322 | Systemic lupus erythematosus | 271/13789 | 0.625035805 | Human Diseases | Immune disease | |
| 2 | map04024 | cAMP signaling pathway | 214/13789 | 0.496849549 | Environmental Information Processing | Signal transduction | |
| 1 | map04068 | FoxO signaling pathway | 131/13789 | 0.641332545 | Environmental Information Processing | Signal transduction | |
| 1 | map05160 | Hepatitis C | 143/13789 | 0.673648034 | Human Diseases | Infectious disease: viral | |
| 2 | map04740 | Olfactory transduction | 246/13789 | 0.57208527 | Organismal Systems | Sensory system | |
| 1 | map04722 | Neurotrophin signaling pathway | 122/13789 | 0.615033849 | Organismal Systems | Nervous system | |
| 2 | map05163 | Human cytomegalovirus infection | 217/13789 | 0.504247741 | Human Diseases | Infectious disease: viral | |
| 1 | map04152 | AMPK signaling pathway | 121/13789 | 0.61199636 | Environmental Information Processing | Signal transduction | |
| 1 | map04550 | Signaling pathways regulating pluripotency of stem cells | 141/13789 | 0.668469846 | Cellular Processes | Cellular community - eukaryotes | |
| 1 | map04120 | Ubiquitin mediated proteolysis | 141/13789 | 0.668469846 | Genetic Information Processing | Folding, sorting and degradation | |
| 1 | map04921 | Oxytocin signaling pathway | 150/13789 | 0.691148654 | Organismal Systems | Endocrine system | |
| 1 | map04932 | Non-alcoholic fatty liver disease (NAFLD) | 150/13789 | 0.691148654 | Human Diseases | Endocrine and metabolic disease | |
| 1 | map04910 | Insulin signaling pathway | 140/13789 | 0.665850312 | Organismal Systems | Endocrine system | |
| 1 | map04371 | Apelin signaling pathway | 136/13789 | 0.655165552 | Environmental Information Processing | Signal transduction | |
| 1 | map03040 | Spliceosome | 136/13789 | 0.655165552 | Genetic Information Processing | Transcription |  |
| 1 | map04360 | Axon guidance | 184/13789 | 0.763783817 | Organismal Systems | Development and regeneration | |
| 1 | map05162 | Measles | 192/13789 | 0.778246961 | Human Diseases | Infectious disease: viral | |
| 1 | map05205 | Proteoglycans in cancer | 208/13789 | 0.804592697 | Human Diseases | Cancer: overview | |
| 1 | map04514 | Cell adhesion molecules (CAMs) | 207/13789 | 0.80304104 | Environmental Information Processing | Signaling molecules and interaction | |
| 1 | map05170 | Human immunodeficiency virus 1 infection | 268/13789 | 0.878555567 | Human Diseases | Infectious disease: viral | |
| 1 | map04060 | Cytokine-cytokine receptor interaction | 275/13789 | 0.885126817 | Environmental Information Processing | Signaling molecules and interaction | |
| 1 | map04010 | MAPK signaling pathway | 294/13789 | 0.901240028 | Environmental Information Processing | Signal transduction | |
| 1 | map04080 | Neuroactive ligand-receptor interaction | 319/13789 | 0.919076609 | Environmental Information Processing | Signaling molecules and interaction | |
