# Supplementary material for: Characterization of Circular RNA Expression Profiles in Colon Specimens of Patients with Slow Transit Constipation
Source: Dis Markers. 2022 Jun 10;2022:3653363. doi: 10.1155/2022/3653363 (PMC9206760; doi:10.1155/2022/3653363)
Supplement: Supplementary 5 — Table S5: top 6 upregulated/downregulated circRNA–miRNA–mRNA regulatory network. [file 3653363.f5.docx]

| **Table S5. Top 6 upregulated/downregulated circRNA-miRNA-mRNA regulatory network** | | |
| --- | --- | --- |
| **Upregulated ciecbase ID** | **miR ID** | **mRNA ID** |
| hsa_circ_0085173 | hsa-miR-582-3p | TFRC |
| hsa_circ_0085173 | hsa-miR-582-3p | AP3M1 |
| hsa_circ_0085173 | hsa-miR-582-3p | ZBTB8A |
| hsa_circ_0085173 | hsa-miR-582-3p | ITGAV |
| hsa_circ_0085173 | hsa-miR-582-3p | TBL1XR1 |
| hsa_circ_0085173 | hsa-miR-582-3p | NUCKS1 |
| hsa_circ_0000542 | hsa-miR-3167 | GRHL1 |
| hsa_circ_0000542 | hsa-miR-3167 | SEMA6A |
| hsa_circ_0000542 | hsa-miR-3167 | TNIP1 |
| hsa_circ_0000542 | hsa-miR-3167 | ATF7IP |
| hsa_circ_0000542 | hsa-miR-3167 | HNRNPA0 |
| hsa_circ_0000542 | hsa-miR-876-5p | TRAPPC8 |
| hsa_circ_0000542 | hsa-miR-876-5p | GRHL1 |
| hsa_circ_0000542 | hsa-miR-876-5p | DYRK1A |
| hsa_circ_0000542 | hsa-miR-876-5p | VTA1 |
| hsa_circ_0000542 | hsa-miR-876-5p | ATF7IP |
| hsa_circ_0000542 | hsa-miR-876-5p | HNRNPA0 |
| hsa_circ_0000542 | hsa-miR-541-5p | ZNF586 |
| hsa_circ_0000542 | hsa-miR-541-5p | LPP |
| hsa_circ_0000542 | hsa-miR-541-5p | PITPNB |
| hsa_circ_0000542 | hsa-miR-541-5p | ONECUT2 |
| hsa_circ_0000542 | hsa-miR-541-5p | ANAPC16 |
| hsa_circ_0000542 | hsa-miR-561-5p | ARFGEF2 |
| hsa_circ_0000542 | hsa-miR-561-5p | HOXA1 |
| hsa_circ_0000542 | hsa-miR-561-5p | SUGP2 |
| hsa_circ_0000542 | hsa-miR-561-5p | REV3L |
| hsa_circ_0000542 | hsa-miR-561-5p | AGO1 |
| hsa_circ_0000542 | hsa-miR-561-5p | NUCKS1 |
| hsa_circ_0030694 | hsa-miR-423-3p | RAP2C |
| hsa_circ_0030694 | hsa-miR-190a-5p | OTUD4 |
| hsa_circ_0030694 | hsa-miR-190a-5p | TRPS1 |
| hsa_circ_0030694 | hsa-miR-190b | MYO5A |
| hsa_circ_0030694 | hsa-miR-19a-3p | QKI |
| hsa_circ_0030694 | hsa-miR-19a-3p | PMEPA1 |
| hsa_circ_0030694 | hsa-miR-19a-3p | LONRF1 |
| hsa_circ_0030694 | hsa-miR-19a-3p | TIA1 |
| hsa_circ_0030694 | hsa-miR-19a-3p | RAB8B |
| hsa_circ_0030694 | hsa-miR-19a-3p | IMPDH1 |
| hsa_circ_0030694 | hsa-miR-370-5p | LONRF3 |
| hsa_circ_0030694 | hsa-miR-370-5p | TENM3 |
| hsa_circ_0030694 | hsa-miR-370-5p | ZMAT3 |
| hsa_circ_0030694 | hsa-miR-370-5p | ADAMTS5 |
| hsa_circ_0030694 | hsa-miR-370-5p | NLN |
| hsa_circ_0030694 | hsa-miR-370-5p | GLRB |
| hsa_circ_0063716 | hsa-miR-138-5p | EIF4EBP1 |
| hsa_circ_0063716 | hsa-miR-138-5p | DUSP16 |
| hsa_circ_0063716 | hsa-miR-138-5p | RARA |
| hsa_circ_0063716 | hsa-miR-138-5p | FOXP4 |
| hsa_circ_0063716 | hsa-miR-138-5p | RMND5A |
| hsa_circ_0063716 | hsa-miR-138-5p | CCND3 |
| hsa_circ_0063716 | hsa-miR-3184-5p | FAM222B |
| hsa_circ_0063716 | hsa-miR-3184-5p | NACC1 |
| hsa_circ_0063716 | hsa-miR-3184-5p | SMG6 |
| hsa_circ_0063716 | hsa-miR-3184-5p | CDC42SE1 |
| hsa_circ_0063716 | hsa-miR-3184-5p | PRKACA |
| hsa_circ_0063716 | hsa-miR-3184-5p | TMEM41A |
| hsa_circ_0063716 | hsa-miR-517a-3p | AREG |
| hsa_circ_0063716 | hsa-miR-517a-3p | IGLL1 |
| hsa_circ_0063716 | hsa-miR-517a-3p | DBN1 |
| hsa_circ_0063716 | hsa-miR-517a-3p | TRPA1 |
| hsa_circ_0063716 | hsa-miR-517a-3p | NFIA |
| hsa_circ_0063716 | hsa-miR-517a-3p | ZNF521 |
| hsa_circ_0063716 | hsa-miR-517b-3p | ZNF521 |
| hsa_circ_0063716 | hsa-miR-517b-3p | DBN1 |
| hsa_circ_0063716 | hsa-miR-517b-3p | NFIA |
| hsa_circ_0063716 | hsa-miR-517b-3p | CBLN2 |
| hsa_circ_0063716 | hsa-miR-517b-3p | TMCC1 |
| hsa_circ_0063716 | hsa-miR-517b-3p | CCDC89 |
| hsa_circ_0063716 | hsa-miR-517c-3p | NUGGC |
| hsa_circ_0063716 | hsa-miR-517c-3p | TMCC1 |
| hsa_circ_0063716 | hsa-miR-517c-3p | TMEM30A |
| hsa_circ_0063716 | hsa-miR-760 | C6orf89 |
| hsa_circ_0063716 | hsa-miR-760 | EIF1 |
| hsa_circ_0063716 | hsa-miR-760 | ZSWIM6 |
| hsa_circ_0063716 | hsa-miR-760 | FBXO8 |
| hsa_circ_0063716 | hsa-miR-760 | ATXN1L |
| hsa_circ_0063716 | hsa-miR-760 | GOLGA7 |

| **Downregulated ciecbase ID** | **miR ID** | **mRNA ID** |
| --- | --- | --- |
| hsa_circ_0016094 | hsa-miR-330-5p | LARP1 |
| hsa_circ_0016094 | hsa-miR-330-5p | TLN1 |
| hsa_circ_0016094 | hsa-miR-330-5p | SLC23A2 |
| hsa_circ_0016094 | hsa-miR-330-5p | MYO1C |
| hsa_circ_0016094 | hsa-miR-330-5p | GPI |
| hsa_circ_0016094 | hsa-miR-330-5p | BSDC1 |
| hsa_circ_0016094 | hsa-miR-556-5p | CDC40 |
| hsa_circ_0016094 | hsa-miR-556-5p | TBC1D4 |
| hsa_circ_0016094 | hsa-miR-556-5p | CSRNP2 |
| hsa_circ_0016094 | hsa-miR-556-5p | RFX3 |
| hsa_circ_0016094 | hsa-miR-2278 | SLC25A28 |
| hsa_circ_0016094 | hsa-miR-2278 | PDP1 |
| hsa_circ_0016094 | hsa-miR-2278 | NRAS |
| hsa_circ_0016094 | hsa-miR-2278 | PATL1 |
| hsa_circ_0016094 | hsa-miR-2278 | CRKL |
| hsa_circ_0016094 | hsa-miR-2278 | CELF1 |
| hsa_circ_0016094 | hsa-miR-432-5p | AXL |
| hsa_circ_0016094 | hsa-miR-432-5p | EIF4G3 |
| hsa_circ_0016094 | hsa-miR-432-5p | GDA |
| hsa_circ_0016094 | hsa-miR-432-5p | TLN1 |
| hsa_circ_0016094 | hsa-miR-432-5p | LASP1 |
| hsa_circ_0016094 | hsa-miR-432-5p | TFPI |
| hsa_circ_0016094 | hsa-miR-183-5p | ITGB1 |
| hsa_circ_0016094 | hsa-miR-183-5p | PPP2CA |
| hsa_circ_0016094 | hsa-miR-183-5p | FRMD6 |
| hsa_circ_0016094 | hsa-miR-183-5p | TCF7L2 |
| hsa_circ_0016094 | hsa-miR-183-5p | TMEM184C |
| hsa_circ_0016094 | hsa-miR-183-5p | EEF2 |
| hsa_circ_0016094 | hsa-miR-760 | C6orf89 |
| hsa_circ_0016094 | hsa-miR-760 | EIF1 |
| hsa_circ_0016094 | hsa-miR-760 | ZSWIM6 |
| hsa_circ_0016094 | hsa-miR-760 | FBXO8 |
| hsa_circ_0016094 | hsa-miR-760 | ATXN1L |
| hsa_circ_0016094 | hsa-miR-760 | GOLGA7 |
| hsa_circ_0071410 | hsa-miR-149-5p | STRADB |
| hsa_circ_0071410 | hsa-miR-149-5p | STARD3 |
| hsa_circ_0071410 | hsa-miR-149-5p | SYT2 |
| hsa_circ_0071410 | hsa-miR-149-5p | BRPF3 |
| hsa_circ_0071410 | hsa-miR-149-5p | MRPL3 |
| hsa_circ_0071410 | hsa-miR-149-5p | SHMT2 |
| hsa_circ_0071410 | hsa-miR-627-5p | EBAG9 |
| hsa_circ_0071410 | hsa-miR-627-5p | ZCCHC17 |
| hsa_circ_0071410 | hsa-miR-627-5p | MAP2K4 |
| hsa_circ_0071410 | hsa-miR-627-5p | FAM78A |
| hsa_circ_0071410 | hsa-miR-627-5p | SKIDA1 |
| hsa_circ_0071410 | hsa-miR-627-5p | RSPO3 |
| hsa_circ_0071410 | hsa-miR-486-5p | SRSF3 |
| hsa_circ_0071410 | hsa-miR-486-5p | BTAF1 |
| hsa_circ_0071410 | hsa-miR-486-5p | ARID4B |
| hsa_circ_0071410 | hsa-miR-486-5p | ST6GALNAC6 |
| hsa_circ_0071410 | hsa-miR-486-5p | PTEN |
| hsa_circ_0071410 | hsa-miR-486-5p | ATXN7L3 |
| hsa_circ_0071410 | hsa-miR-769-5p | TFAM |
| hsa_circ_0071410 | hsa-miR-769-5p | SAR1B |
| hsa_circ_0071410 | hsa-miR-769-5p | CERCAM |
| hsa_circ_0071410 | hsa-miR-769-5p | CDK1 |
| hsa_circ_0071410 | hsa-miR-769-5p | SET |
| hsa_circ_0071410 | hsa-miR-769-5p | CCNL1 |
| hsa_circ_0071410 | hsa-miR-6512-3p | ELP6 |
| hsa_circ_0071410 | hsa-miR-6512-3p | P2RX6 |
| hsa_circ_0071410 | hsa-miR-6512-3p | CYB5R2 |
| hsa_circ_0071410 | hsa-miR-6512-3p | ZNF821 |
| hsa_circ_0071410 | hsa-miR-6512-3p | FAM180A |
| hsa_circ_0071410 | hsa-miR-6512-3p | ODF3L2 |
| hsa_circ_0071410 | hsa-miR-9-5p | ONECUT2 |
| hsa_circ_0071410 | hsa-miR-9-5p | POU2F1 |
| hsa_circ_0071410 | hsa-miR-9-5p | TRPM7 |
| hsa_circ_0071410 | hsa-miR-9-5p | LDLRAP1 |
| hsa_circ_0071410 | hsa-miR-9-5p | CTNNA1 |
| hsa_circ_0071410 | hsa-miR-9-5p | MTHFD2 |
| hsa_circ_0063878 | hsa-miR-216a-5p | TMEM161B |
| hsa_circ_0063878 | hsa-miR-216a-5p | BMP10 |
| hsa_circ_0063878 | hsa-miR-216a-5p | PROKR2 |
| hsa_circ_0063878 | hsa-miR-216a-5p | HNF4G |
| hsa_circ_0063878 | hsa-miR-216a-5p | RANBP10 |
| hsa_circ_0063878 | hsa-miR-216a-5p | HOOK1 |
| hsa_circ_0063878 | hsa-miR-30a-3p | HMGN4 |
| hsa_circ_0063878 | hsa-miR-30a-3p | ZNHIT6 |
| hsa_circ_0063878 | hsa-miR-30a-3p | GREM1 |
| hsa_circ_0063878 | hsa-miR-30a-3p | HLA-B |
| hsa_circ_0063878 | hsa-miR-30a-3p | LYRM2 |
| hsa_circ_0063878 | hsa-miR-30a-3p | UNG |
| hsa_circ_0063878 | hsa-miR-30d-3p | SH3D19 |
| hsa_circ_0063878 | hsa-miR-30d-3p | CCNT2 |
| hsa_circ_0063878 | hsa-miR-30d-3p | TMEM87A |
| hsa_circ_0063878 | hsa-miR-30d-3p | ATP9B |
| hsa_circ_0063878 | hsa-miR-30d-3p | ZMAT3 |
| hsa_circ_0063878 | hsa-miR-30d-3p | SPOPL |
| hsa_circ_0063878 | hsa-miR-30e-3p | INIP |
| hsa_circ_0063878 | hsa-miR-30e-3p | GFPT1 |
| hsa_circ_0063878 | hsa-miR-30e-3p | PTEN |
| hsa_circ_0063878 | hsa-miR-30e-3p | PHACTR2 |
| hsa_circ_0063878 | hsa-miR-30e-3p | GALNT1 |
| hsa_circ_0063878 | hsa-miR-30e-3p | CAP2 |
| hsa_circ_0063878 | hsa-miR-3120-3p | LCTL |
| hsa_circ_0063878 | hsa-miR-3120-3p | EID1 |
| hsa_circ_0063878 | hsa-miR-3120-3p | TLR8 |
| hsa_circ_0063878 | hsa-miR-3120-3p | TMEM61 |
| hsa_circ_0063878 | hsa-miR-3120-3p | SCAF4 |
| hsa_circ_0063878 | hsa-miR-3120-3p | SALL1 |
| hsa_circ_0063878 | hsa-miR-3193 | IFITM10 |
| hsa_circ_0063878 | hsa-miR-3193 | SHOX2 |
| hsa_circ_0063878 | hsa-miR-3193 | HTRA4 |
| hsa_circ_0063878 | hsa-miR-3193 | NME4 |
| hsa_circ_0063878 | hsa-miR-3193 | SURF6 |
| hsa_circ_0063878 | hsa-miR-3193 | TLDC2 |
| hsa_circ_0004214 | hsa-miR-6763-5p | PRX |
| hsa_circ_0004214 | hsa-miR-6763-5p | SYNGAP1 |
| hsa_circ_0004214 | hsa-miR-6763-5p | OAF |
| hsa_circ_0004214 | hsa-miR-6763-5p | KSR2 |
| hsa_circ_0004214 | hsa-miR-6763-5p | NACC1 |
| hsa_circ_0004214 | hsa-miR-6763-5p | ELK1 |
| hsa_circ_0004214 | hsa-miR-382-5p | AMOTL2 |
| hsa_circ_0004214 | hsa-miR-526b-5p | TPM4 |
| hsa_circ_0004214 | hsa-miR-526b-5p | PM20D2 |
| hsa_circ_0004214 | hsa-miR-526b-5p | GATA6 |
| hsa_circ_0004214 | hsa-miR-526b-5p | ABCF2 |
| hsa_circ_0004214 | hsa-miR-193a-5p | COL1A1 |
| hsa_circ_0004214 | hsa-miR-193a-5p | FADS1 |
| hsa_circ_0004214 | hsa-miR-193a-5p | IGF2 |
| hsa_circ_0004214 | hsa-miR-379-5p | SLC20A1 |
| hsa_circ_0004214 | hsa-miR-3529-5p | C5orf51 |
| hsa_circ_0004214 | hsa-miR-3529-5p | EIF4G2 |
| hsa_circ_0004214 | hsa-miR-3529-5p | CDKN2AIP |
| hsa_circ_0004214 | hsa-miR-3529-5p | MTMR2 |
| hsa_circ_0004214 | hsa-miR-3529-5p | UBE2E3 |
| hsa_circ_0004214 | hsa-miR-3529-5p | PXT1 |
